# Lone Pair Rotation and Bond Heterogeneity Leading to Ultralow Thermal Conductivity in Aikinite

**DOI:** 10.1021/jacs.3c02536

**Published:** 2023-04-13

**Authors:** Virginia Carnevali, Shriparna Mukherjee, David J. Voneshen, Krishnendu Maji, Emmanuel Guilmeau, Anthony V. Powell, Paz Vaqueiro, Marco Fornari

**Affiliations:** † Department of Physics and Science of Advanced Materials Program, Central Michigan University, Mt. Pleasant, Michigan 48859, United States; ‡ Department of Chemistry, University of Reading, Whiteknights, Reading RG6 6DX, U.K.; § ISIS Pulsed Neutron and Muon Source, Rutherford Appleton Laboratory, Chilton, Didcot OX11 0QX, Oxon, U.K.; ∥ Department of Physics, Royal Holloway University of London, Egham TW20 0EX, U.K.; ⊥ CRISMAT, CNRS, Normandie Univ, ENSICAEN, UNICAEN, Caen 14000, France

## Abstract

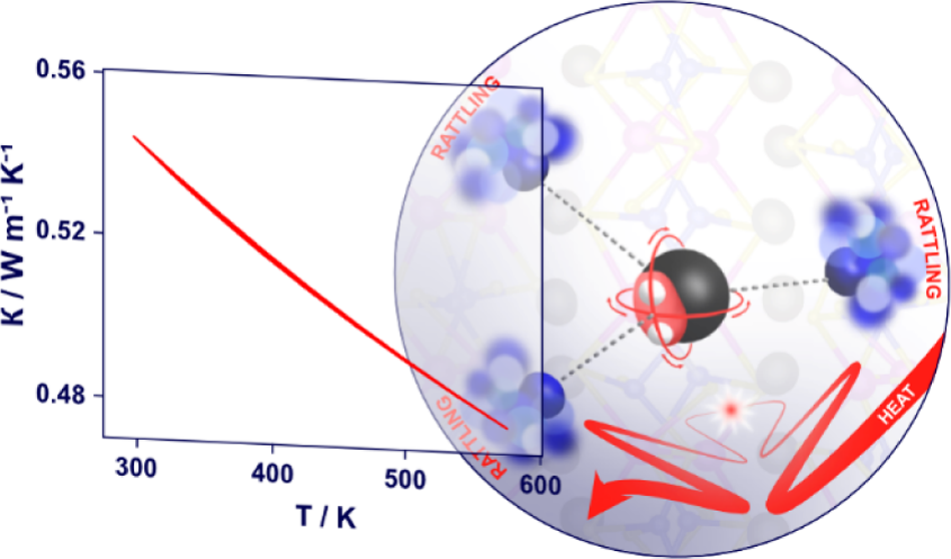

Understanding the relationship between the crystal structure, chemical bonding, and lattice dynamics is crucial for the design of materials with low thermal conductivities, which are essential in fields as diverse as thermoelectrics, thermal barrier coatings, and optoelectronics. The bismuthinite-aikinite series, Cu_1–*x*_□_*x*_Pb_1–*x*_Bi_1+*x*_S_3_ (0 ≤ *x* ≤ 1, where □ represents a vacancy), has recently emerged as a family of *n*-type semiconductors with exceptionally low lattice thermal conductivities. We present a detailed investigation of the structure, electronic properties, and the vibrational spectrum of aikinite, CuPbBiS_3_ (*x* = 0), in order to elucidate the origin of its ultralow thermal conductivity (0.48 W m^–1^ K^–1^ at 573 K), which is close to the calculated minimum for amorphous and disordered materials, despite its polycrystalline nature. Inelastic neutron scattering data reveal an anharmonic optical phonon mode at *ca.* 30 cm^–1^, attributed mainly to the motion of Pb^2+^ cations. Analysis of neutron diffraction data, together with *ab-initio* molecular dynamics simulations, shows that the Pb^2+^ lone pairs are rotating and that, with increasing temperature, Cu^+^ and Pb^2+^ cations, which are separated at distances of *ca*. 3.3 Å, exhibit significantly larger displacements from their equilibrium positions than Bi^3+^ cations. In addition to bond heterogeneity, a temperature-dependent interaction between Cu^+^ and the rotating Pb^2+^ lone pair is a key contributor to the scattering effects that lower the thermal conductivity in aikinite. This work demonstrates that coupling of rotating lone pairs and the vibrational motion is an effective mechanism to achieve ultralow thermal conductivity in crystalline materials.

## Introduction

Thermal transport is of paramount importance for a broad range of technological applications. For instance, thermally insulating materials can be used to prevent heat from damaging critical components,^1^ and the performance, safety, and lifetime of batteries in electric vehicles can be improved with optimized thermal management.^2^ Finding materials that combine the desired thermal conductivity with other properties required for specific technological applications can be exceptionally challenging. This is the case for thermoelectric devices, which enable the conversion of a temperature difference into electrical power. For thermoelectric applications, materials with low thermal conductivity are required in order to limit parasitic heat transfer. However, these materials also need to be excellent electrical conductors; this is a conflicting requirement because heat is transported by electrons as well as by phonons. A variety of extrinsic and intrinsic strategies have been proposed in order to minimize phonon transport (which determines the lattice thermal conductivity, κ_L_) in thermoelectric materials.^3^ While extrinsic effects, such as multiscale hierarchical structuring,^4^ grain-boundary engineering,^5^ and nanoprecipitates,^6^ are effective at lowering κ_L_, they can also adversely influence other properties, such as mechanical and thermal stability, as well as the charge-carrier mobility. Recent research efforts have focused on intrinsic mechanisms that reduce κ_L_ by tuning the structure and bonding of materials. These mechanisms include complex crystal structures with a large number of atoms per unit cell,^7^ order–disorder phenomena,^8,9^ liquid-like ionic mobility (phonon-liquid-electron-crystal),^10^ rattling,^11^ resonant bonding,^12^ anharmonicity induced by lone pairs,^13^ and bonding heterogeneity.^14,15^

The efficiency of thermoelectric energy recovery is related to the figure of merit,  (where σ, *S*, *T*, κ_*e*_, and κ_L_ are the electrical conductivity, the Seebeck coefficient, the operating temperature, and the electronic and lattice components of the thermal conductivity, respectively) of the thermoelectric materials found in the device.^16^ Among the chalcogenides, sulfide-based minerals are attractive as potential thermoelectric materials due to the large terrestrial abundance and availability of sulfur when compared to selenium and tellurium.^17^ A feature common to many of the best performing thermoelectric sulfides is their low thermal conductivity, the origin of which is not fully understood. Promising *p*-type sulfide minerals, with a thermoelectric figure of merit, *ZT*, approaching unity at moderate temperatures and low thermal conductivity, include tetrahedrites, Cu_12+*x*_Sb_4_S_13_^18,19^ and Cu_12*-x*_*M*_*x*_Sb_4_S_13_ (*M* = Zn, Ni),^20−22^ colusites, Cu_26_*T*_2_*M*_6_S_32_ (*T* = Cr, Mo and W, and *M* = Ge and Sn)^23−26^ and bornite, Cu_5_FeS_4_.^27^ By contrast, progress on the corresponding *n*-type sulfides has been limited,^28^ with several bismuth-containing sulfides being among the best *n*-type candidates for thermoelectric applications at moderate temperatures. For instance, bismuthinite, Bi_2_S_3_, reaches *ZT* ≈ 0.6 at 773 K when doped with chlorine,^29^ while CdPb_2_Bi_4_S_9_, which is a member of the pavonite-homologous series, M_*n*+1_Bi_2_Q_*n*+5_ (*n* = 4), exhibits a figure of merit, *ZT* = 0.53 at 775 K.^30^

**Figure 1:**
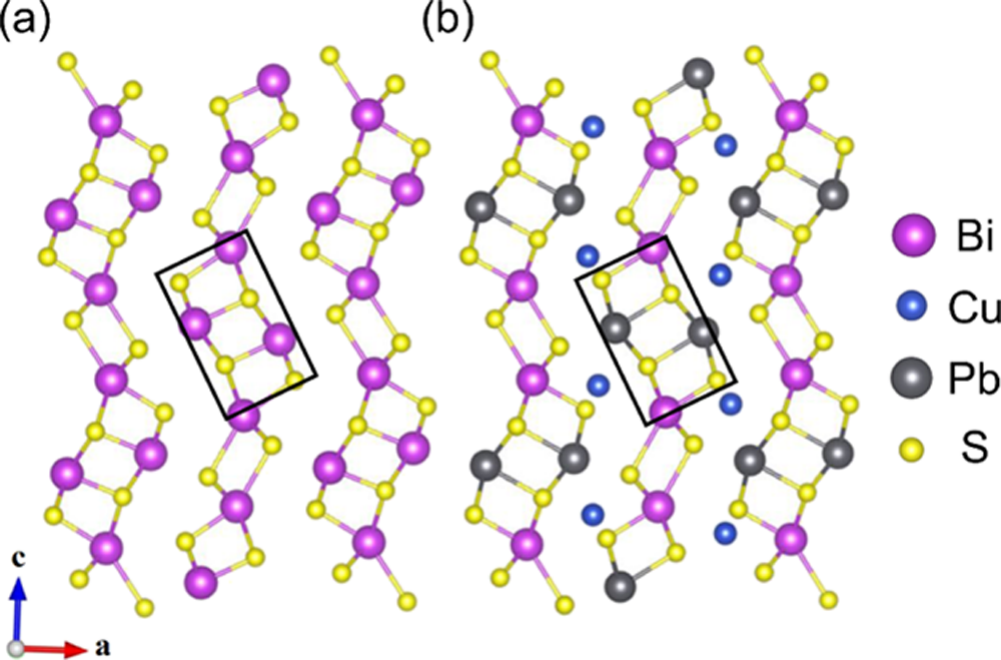
Comparison of the structures of (a) Bi_2_S_3_ and (b) CuPbBiS_3_ (space group, *Pnma*); the black rectangle highlights the Bi_4_S_6_ ribbons in Bi_2_S_3_ and the Bi_2_Pb_2_S_6_ ribbons in aikinite.

Very recently, it has been reported that the quaternary sulfide CuPbBi_5_S_9_ exhibits ultralow thermal conductivity, κ ≈ 0.5 W m^–1^ K^–1^, and can reach *ZT* = 0.43 at 700 K upon doping.^31,32^ This material is a member (*x* = 2/3) of the bismuthinite-aikinite series, Cu_1–*x*_□_*x*_Pb_1–*x*_Bi_1+*x*_S_3_ (0 ≤ *x* ≤ 1), all of which exhibit closely related crystal structures (Figure 1).^33,34^ The structure of bismuthinite, Bi_2_S_3_ (*x* = 1), which is highly anisotropic, contains Bi_4_S_6_ ribbons arranged in a herringbone pattern. In aikinite (*x* = 0), half of the Bi^3+^ cations are replaced with Pb^2+^, with Cu^+^ cations filling the tetrahedral holes between the Bi_2_Pb_2_S_6_ ribbons. In the bismuthinite-aikinite series, the aikinite end member (*x* = 0), as well as kruptaite, CuPbBi_3_S_6_ (*x* = 0.5), adopt unit cells based on the archetype illustrated in Figure 1b. In natural specimens, minerals with intermediate compositions exhibit superstructures based on the ordered intergrowth of blocks of bismuthinite, aikinite, and kruptaite.^35^ However, cation ordering is extremely slow, and synthetic materials with intermediate compositions adopt a copper-deficient aikinite structure, which contains disordered vacancies at the tetrahedral copper site.^36^

Despite the large number of minerals in the aikinite-bismuthinite series, little is known about the electrical and thermal transport properties of these materials, with the exception of those of Bi_2_S_3_ and CuPbBi_5_S_9_,^29,31^ which are *n-*type semiconductors. Here, we present a detailed study of the structure and transport properties of aikinite, CuPbBiS_3_, from experimental and theoretical points of view. We demonstrate that CuPbBiS_3_ is a crystalline *p*-type semiconductor with ultralow lattice thermal conductivity, close to the minimum lattice thermal conductivity calculated on the basis of Cahill’s model^37^ for amorphous and disordered materials. Although neutron diffraction data indicate that in the crystal structure of aikinite the Cu^+^, Pb^2+^, and Bi^3+^ cations are fully ordered, our sound velocity measurements reveal that, at room temperature, the phonon mean-free-path is only *ca.* 5 Å, which is approximately twice that of the interatomic spacing. The temperature dependence of the vibrational spectrum has been investigated by *ab-initio* molecular dynamics (AIMD), while inelastic neutron scattering (INS) data have been exploited to estimate the lifetime of the low-frequency Pb^2+^ mode, which is only *ca.* 0.4 ps. Our analysis of the calculated and experimental vibrational density of states (vDOS), which are in excellent agreement, provides clear evidence for the presence of Cu^+^ rattling-like modes, together with anharmonic low-energy modes arising from weakly bonded Pb^2+^ cations. *Ab-initio* molecular dynamics simulations reveal that the intrinsic mechanism responsible for the ultralow thermal conductivity in aikinite entails the cooperative interaction between the rotating lone pair on the Pb^2+^ cations and the Cu^+^ cations.

## Experimental and Computational Section

### Material Synthesis

CuPbBiS_3_ was prepared by mechanical alloying of stoichiometric amounts of Cu (Sigma-Aldrich, powder 425 μm, 99.5%), Pb (Goodfellow, rods 3.2 mm dia., 99.95%), Bi (Alfa Aesar, needles, 99.99%), and S (Sigma-Aldrich, flakes, 99.99%). The reagents were handled under an Ar atmosphere, inside a glovebox. Prior to loading the elements into a 45 mL ball-milling jar made of stainless steel, the Pb rods were cut into small pieces, and Bi needles were ground into a powder using a pestle and mortar. Eighteen stainless-steel balls, each with a diameter of 10 mm and a weight of 4 g, were added to the ball-milling jar. A powder-to-ball weight ratio of 1:12 was used. Milling was carried out using a Fritsch Pulverisette 6 Planetary Ball Mill at 500 rpm for 60 h and stopped for 10 min every 10 min of milling. Following milling, the resulting powder was sealed into an evacuated (<10^–4^ mbar) fused-silica ampoule. The sealed ampoule was heated to 573 K (at a rate of 1 K min^–1^), held for 48 h at this temperature, and subsequently cooled to room temperature (at a rate of 1 K min^–1^). The annealed powder was hand-ground in air and consolidated into a densified pellet of *ca.* 13 mm diameter by hot pressing under N_2_ at 473 K under 80 MPa for 1 h. The pressure was released, and the hot press was then cooled down for 1 h to room temperature. The density of the hot-pressed pellet was determined by the Archimedes’ method, using an AE Adam PW 184 balance. The pellet has a density greater than 96% of the crystallographic density of aikinite.

### X-ray and Neutron Diffraction Data Collection and Analysis

Powder X-ray diffraction data were collected on a Bruker D8 Advance powder X-ray diffractometer equipped with a LynxEye detector and operating with monochromatic Cu Kα1 (λ = 1.54046 Å) radiation. Data collections of 8 h over the range 10 ≤ 2θ/° ≤ 120 were used. Lattice parameters were determined by the Rietveld method, carried out using GSAS.^38^ A shifted Chebyschev polynomial with ten coefficients was used to model the background, and a pseudo-Voigt function was used to model the peak shape. High-resolution neutron powder diffraction data were collected on the time-of-flight POWGEN beamline^39^ at the Spallation Neutron Source (Oak Ridge National Laboratory, US). Data were collected at room temperature for 3 h using the center wavelength setting of 1.5 Å, with a *d*-spacing coverage of 0.5 ≤ *d*/Å ≤ 11.8. The powder sample was loaded into a vanadium can with an 8 mm inner diameter and sealed with a copper gasket and aluminum lid. Rietveld refinements using neutron data were carried out using GSAS-II.^40^ A logarithmic interpolation function with twenty terms was used to model the background contribution. Lattice parameters, atomic coordinates, profile parameters, and the phase fraction for both aikinite and PbS were refined. A single isotropic atomic displacement parameter (*U*_iso_) was used for all the sulfur atoms in the aikinite phase and refined along with those of copper, lead, and bismuth atoms. Different structural models for the aikinite phase were explored, as detailed in the results section.

### Optical Measurements

A diffuse reflectance measurement was carried out over the wavelength range 200 ≤ λ/nm ≤ 2500 with a step size of 1 nm using an Agilent Cary 7000 spectrophotometer equipped with a diffuse reflectance accessory. The reflectance data were transformed into the corresponding absorption spectra using the Kubelka–Munk function, , where *R*_∞_ is the reflectance of an infinitely thick specimen and *K* and *S* are the absorption and scattering coefficients, respectively. The band gap (*E*_g_) was estimated using the Tauc method,^41^ from the linear fit of (*F*(*R*_∞_)*h*ν)^1/*n*^ = *A*(*h*ν – *E*_g_) vs. *h*ν, where *A* is a proportionality constant, *h* is Planck’s constant, ν is the photon frequency, and *n* = 1/2 or 2 for direct and indirect transitions, respectively.

### Thermal and Electrical Property Measurements

Differential scanning calorimetry (DSC) data over the temperature range 300 ≤ *T*/K ≤ 575 and a heating rate of 10 K min^–1^ were collected under a flowing N_2_ atmosphere using a TA-Q2000 DSC instrument. Thermogravimetric analysis (TGA) was carried out over the temperature range 300 ≤ *T*/K ≤ 970 using a TA-TGA Q50 under a N_2_ atmosphere; a heating rate of 10 K min^–1^ was used. The electrical conductivity and Seebeck coefficient were measured simultaneously using a Linseis LSR 3 instrument, using a 4-probe configuration, under a helium atmosphere. Measurements were carried out on pellets with a diameter of 12.7 mm and thickness of ∼1.5–2 mm. The electrical conductivity and Seebeck coefficient were measured over the temperature range of 423 ≤ *T*/K ≤ 573. A current of 20 mA was used for the conductivity measurements, and a maximum gradient of 50 K was maintained between the upper and lower electrodes for the measurement of the Seebeck coefficient. The instrument was calibrated using a constantan reference. Thermal diffusivity (*D*) measurements were made on graphite-coated circular pellets with a diameter of 12.7 mm and a thickness of ∼1.5–2 mm, using a Netzsch LFA 447 NanoFlash system, over the temperature range of 273 ≤ *T*/K ≤ 573. Data were analyzed using Cowan’s model with a pulse correction applied. The thermal conductivity (κ) was then calculated from the relation, κ = *DdC*_p_, where *d* is the density of the material and *C*_p_ is the specific heat capacity. The Dulong-Petit limit for *C*_p_, which for CuPbBiS_3_ is 0.259 J g^–1^ K^–1^, has been used. The uncertainties in the values of the electrical resistivity, Seebeck coefficient, and thermal conductivity are 5, 5, and 10% respectively. The calculations of the minimum thermal conductivity are described in the Supporting Information. The Lorenz number *L* was determined using the relation *L* = 1.5 + exp(−|*S*|/116),^42^ where *S* is the temperature-dependent Seebeck coefficient. Using the Wiedemann–Franz relation, the electronic part of thermal conductivity κ_e_ was calculated. Hall effect measurements, to determine the charge carrier concentration, were carried out at room temperature using a physical properties measurement system (PPMS, Quantum Design) under applied magnetic fields of up to 9 T.

### Sound Velocity Measurements

The transverse (*v*_T_) and longitudinal velocities (*v*_L_) of sound were measured on a disc-shaped sample of *ca.* 12.5 mm diameter and *ca*. 2 mm thickness using an Olympus ultrasonic flaw detector (model 38DL plus) with a transducer frequency of 5 MHz.

### Inelastic Neutron Scattering Data

INS data were collected using the LET spectrometer (ISIS Neutron and Muon Source, UK).^43^ The powder sample was loaded into an annular aluminum can. Data were collected at four temperatures, i.e., 10, 100, 200, and 300 K. The LET choppers were set up to use incident energies of 24.93, 9.01, 4.60, and 2.79 meV. Identical measurements were carried out for the empty aluminum can, and the instrumental background was subtracted using the Mantid package.^44,45^ The INS data were integrated from 3 to 5 Å^–1^ in *Q*-space. The neutron-weighted phonon density of states was normalized using a custom Python script. The phonon energies were analyzed using the DAVE package.^46^ Peaks were modeled using individual Gaussian functions.

### Electronic Bands and Transport

*Ab-initio* electronic calculations were performed using Quantum ESPRESSO^47^ as incorporated in the high-throughput infrastructure AFLOWπ.^48^ We used ultrasoft PBE pseudopotentials, well-converged basis sets corresponding to an energy cutoff of 60 Ry for the wave functions and 600 Ry for the charge density, and the ACBN0 approach^49^ to self-consistently determine the values for the Hubbard corrections for each atomic species of the material (U(Pb) = 0.003 eV, U(Cu) = 3.403 eV, U(Bi) = 0.014 eV, and U(S) = 1.589 eV). Hubbard corrections were applied to the 3d orbitals of Cu, the 6p orbitals of Pb and Bi, and the 3p orbitals of S. Spin–orbit coupling was included in the calculation. To integrate over the Brillouin zone, we used a 4 × 8 × 4 Monkhorst–Pack k-point mesh.^50^ The optimized theoretical lattice parameters used for the *ab-initio* simulations are *a* = 11.943 Å, *b* = 4.058 Å, and *c* = 11.321 Å. The effective masses have been computed with the method developed by Supka *et al.*(51)

### Born-Oppenheimer *Ab-Initio* Molecular Dynamics

AIMD simulations with the mixed Gaussian and plane wave (GPW) method as implemented in the CP2K package^52^ were performed to compute the vibrational and structural properties of aikinite as a function of temperature. Valence electrons were expanded as a double-ζ Gaussian basis set with polarization functions (DZVP).^53^ The energy cutoff for the electron density expansion in the GPW method was 400 Ry. The temperature was controlled by the velocity-rescaling thermostat of Bussi *et al.*(54) with a time constant of 1.0 fs. The system was first equilibrated to 300 K for 10 ps in the isothermal-isobaric ensemble (*NPT*) with *P* = 1 atm. The system was then equilibrated in the microcanonical (NVE) ensemble, and statistics were gathered for the last 10 ps of the production run. Maximally localized Wannier functions (MLWF)^55^ and their centers (MLWFC) were obtained using CP2K, minimizing the MLWF spreads as explained by Berghold *et al.*(56) The Pb^2+^ lone pair dynamics has been characterized through the rotational time correlation function (TCF)^57^
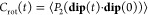
where **dip**(*t*) is the Pb^2+^ MLWFC dipole moment at time *t*, and *P*_2_(*x*) is the second-order Legendre polynomial.

### Vibrational Properties

Phonon dispersions and the vDOS for aikinite were reported by Maji *et al.*(26) and serve as a starting point for the computation of the lattice thermal conductivity in the quasi-harmonic approximations.^58,59^ The transverse and longitudinal sound velocities have been derived in two different ways: from the phonon dispersion and from the computed elastic constants (see Supporting Information). All AIMD simulations were performed at the Γ-point in a 2 × 5 × 2 supercell. The lattice parameters of the triclinic simulation box were allowed to relax for 10 ps in the *NPT* ensemble with *P* = 1 atm. The system was then equilibrated over 5 ps in the isothermal-isochoric ensemble (*NVT*) using the supercell volume obtained by the *NPT* runs; 20 ps of simulation were used to estimate temperature-dependent effects at 100, 200, and 300 K. The vDOS, *D*(ω), was calculated as a Fourier transform of the velocity autocorrelation function (VACF) as
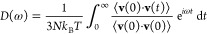
where ⟨**v**(0)·**v**(*t*)⟩ is the VACF computed over the production’s run trajectory, ω is the frequency, *N* is the number of atoms, *k*_B_ is the Boltzmann constant, and *T* is the absolute temperature. We found good agreement between the Quantum ESPRESSO and the CP2K results when comparisons were possible. From the calculated vDOS, we have also computed the Helmholtz free energy *F*, the internal energy *E*, the entropy *S*, and the specific heat *C*_v_ at zero pressure (Supporting Information, Figure S1).

## Results and Discussion

### Crystal Structure and Bonding

Powder X-ray diffraction data collected at room temperature can be indexed on an orthorhombic unit cell (space group *Pnma*); Rietveld refinement yielded lattice parameters of *a* = 11.6136(2) Å, *b* = 4.0433(1) Å, and *c* = 11.3675(2) Å, which are in good agreement with those previously reported for aikinite (CuPbBiS_3_).^60^ This refinement also indicates that the sample is essentially a single phase (*ca.* 99 wt %), with only trace amounts of PbS (*ca.* 1 wt %) present. Changes to the synthetic procedures, including sealed-tube synthesis instead of ball milling, different hot pressing temperatures, and replacement of elemental Pb with PbS, were attempted to remove the traces of PbS. These attempts did not increase the weight percentage of aikinite above 99%.

According to the X-ray structural model for aikinite,^61^ in which differences in bond lengths were used to allocate crystallographic sites to bismuth and lead, the Pb^2+^ and Bi^3+^ cations are fully ordered into two distinct crystallographic sites, M1 (0.332, 1/4, 0.448) and M2 (0.0185, 1/4, 0.681), respectively. As it is impossible to distinguish between isoelectronic Pb^2+^ and Bi^3+^ by X-ray diffraction methods, we collected room-temperature powder neutron diffraction data, which provides contrast between this pair of elements (*b*_Pb_ = 9.4; *b*_Bi_ = 8.5 fm). Rietveld refinements using neutron data were carried out considering three alternative scenarios for the cation distribution: (1) all Pb^2+^ cations located on M1 and all Bi^3+^ cations on M2, (2) all Pb^2+^ cations located on M2 and all Bi^3+^ cations on M1, and (3) Pb^2+^ and Bi^3+^ cations disordered between the M1 and M2 sites.

**Figure 2:**
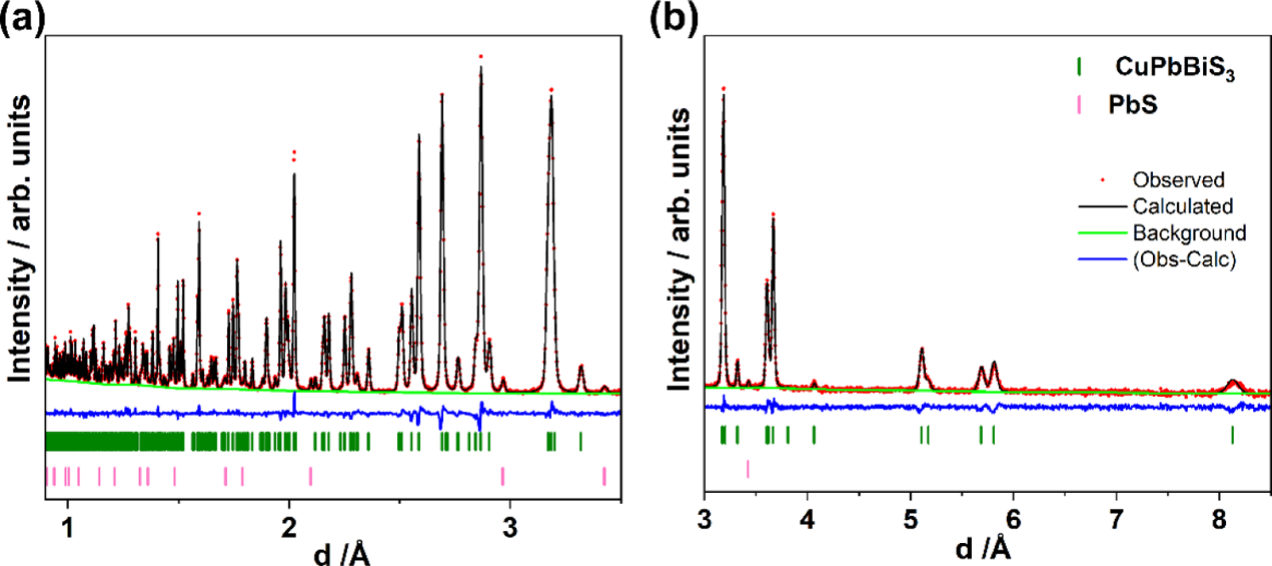
Rietveld refinement using neutron diffraction data for CuPbBiS_3_; (a) data over the range 0.9 ≤ *d*/Å ≤ 3.5 and (b) 3 ≤ *d*/Å ≤ 8.5. The refinement corresponds to structural model (1), in which all Pb^2+^ cations are located on the M1 site and all Bi^3+^ cations on the M2 site.

Clear mismatches in intensities are observed when the Pb^2+^ cations are located on the M2 site and the Bi^3+^ cations are located on the M1 site (Supporting Information, Figure S2a,b), leading to a significantly higher *R*_wp_ factor. Structural disorder associated with the M1 and M2 sites (model (3)) was introduced by refining the site occupancy factors (SOFs), with the constraint that each site remained fully occupied and that the overall stoichiometry was maintained. This, however, did not improve *R*_wp_ (Supporting Information, Figure S2c,d). The lowest value of *R*_wp_ is found when Pb^2+^ cations and Bi^3+^ cations are fully ordered on M1 and M2 sites, respectively (Figure 2). Refined parameters are presented in Table 1, while selected bond lengths and the corresponding bond valence sums are presented in the Supporting Information, Tables S1–S4. The latter are consistent with the formal oxidation states of Pb^2+^, Bi^3+^, and Cu^+^.

**Table 1:** Refined Parameters for CuPbBiS_3_ (Space Group *Pnma*) Obtained from a Rietveld Refinement Using Neutron Diffraction Data Collected at Room Temperature^a^

atom	Wyckoff site	*x*	*y*	*z*	SOF	*U*_iso_ (Å^2^)
Pb (M1)	4*c*	0.33288(7)	0.25	0.48930(7)	1	2.29(2)
Bi (M2)	4*c*	0.01707(6)	0.25	0.68216(7)	1	1.43(2)
Cu	4*c*	0.23464(8)	0.25	0.20908(8)	1	2.10(2)
S1	4*c*	0.0470(2)	0.25	0.1391(2)	1	1.28(3)
S2	4*c*	0.3790(2)	0.25	0.0558(2)	1	1.28(3)
S3	4*c*	0.2143(2)	0.25	0.8013(2)	1	1.28(3)

a *R*_wp_ = 2.87% and GOF = 2.39. Lattice parameters: *a* = 11.61722(7) Å, *b* = 4.044072(23) Å, and *c* = 11.37165(7) Å. Weight fraction: CuPbBiS_3_ = 98.99(5)% and PbS = 1.01(5)%.

**Figure 3:**
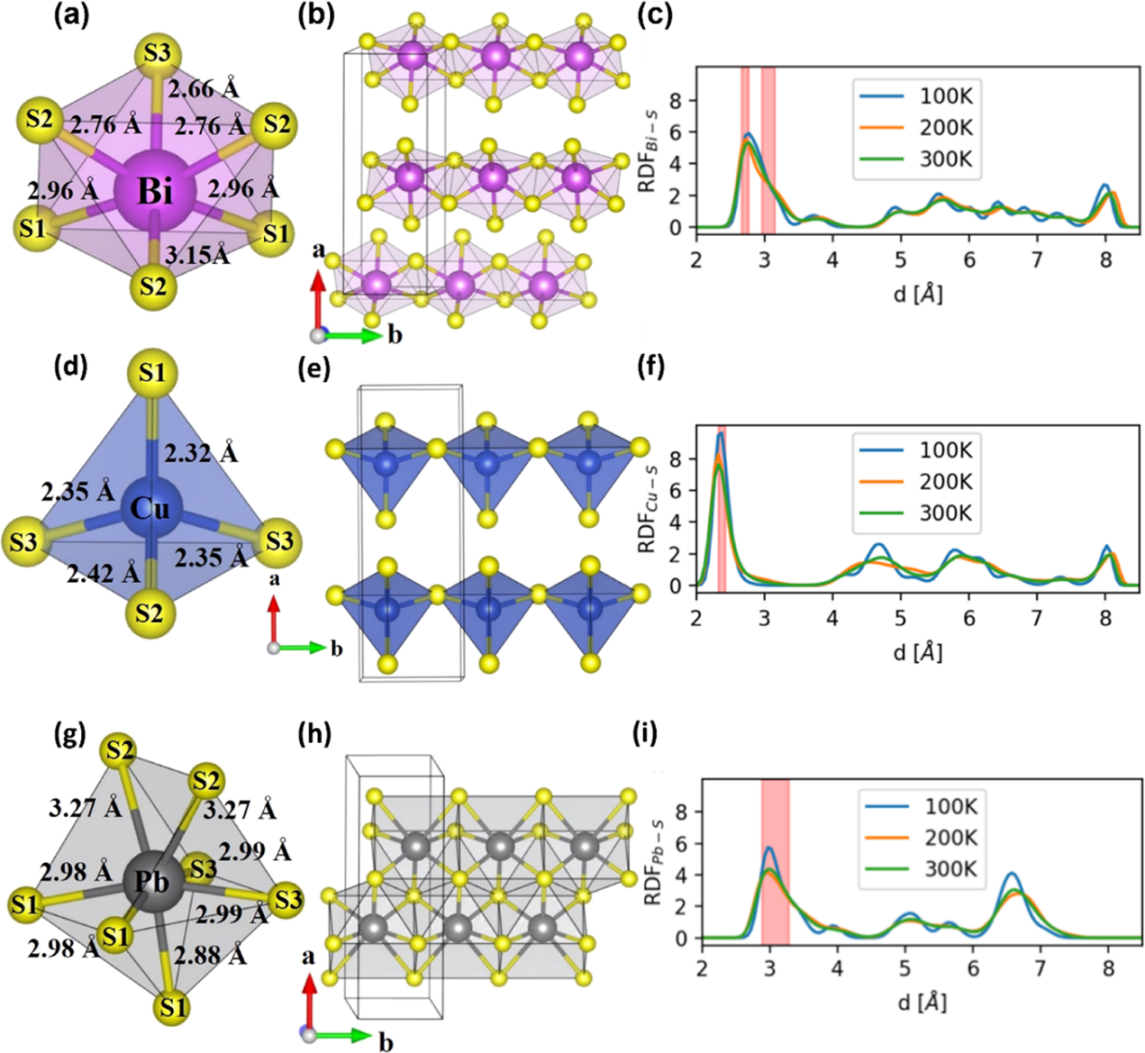
(a) Distorted octahedral coordination of Bi^3+^ (purple sphere) with sulfur (yellow sphere). (b) One-dimensional [BiS_4_]^−^ chains directed along the *b*-axis. (c) Bi–S RDF computed from the *NVT ab initio* molecular dynamics trajectory. (d) Distorted tetrahedral coordination of Cu^+^ (blue sphere) with sulfur. (e) Chains of corner-sharing [CuS_4_]^7–^ tetrahedra along the *b*-axis. (f) Cu–S RDF computed from the *NVT ab initio* molecular dynamics trajectory. (g) Capped octahedral coordination of Pb^2+^ (gray sphere) with sulfur. (h) Ribbons, of stoichiometry [PbS_3_]^−^, directed along the *b*-axis. (i) Pb–S RDF computed from the *NVT ab initio* molecular dynamics trajectory. Legend for the RDF plots: blue, orange, and green lines correspond to simulations at 100, 200, and 300 K, respectively; the red shaded area highlights the experimental distances.

**Figure 4:**
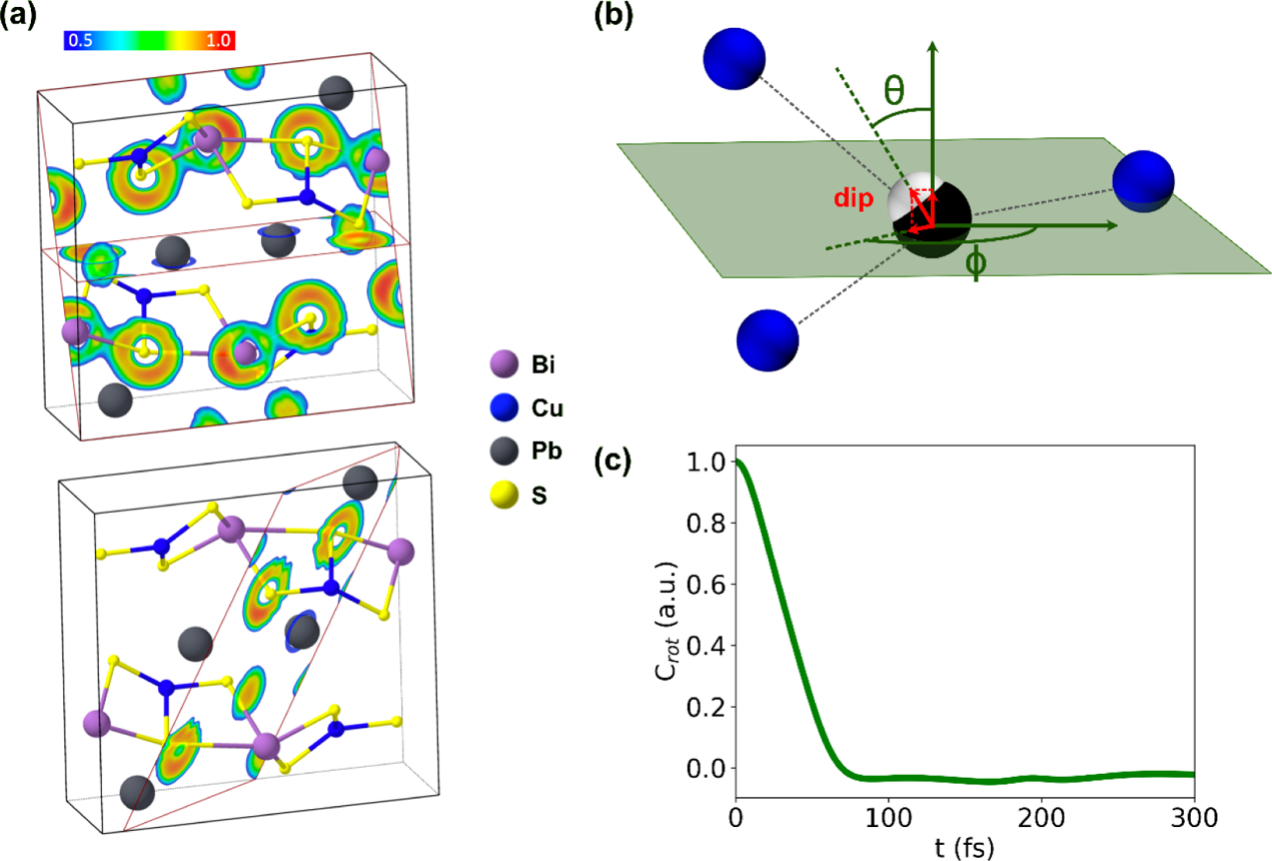
(a) Contour of the ELF on the (011) and (001) (top), and (−211) (bottom) planes. Values for the ELF range between 0 and 1: ELF = 0.5 (blue contours) indicates free electron behavior, and ELF = 1.0 (red contour) indicates perfect localization. Values smaller than 0.5 are less significant and usually point to small local electron densities. (b) *Ab initio* molecular dynamics (AIMD) snapshot highlighting the Pb^2+^ (black)—Cu^+^ (blue) coordination environment. The white sphere indicates the center of the maximally localized Wannier functions (MLWFC) associated with the Pb^2+^ lone pair. The dipole moment points from the Pb^2+^ position to the MLWFC. The θ and ϕ angles are used to indicate the dipole orientation; ϕ is in the plane in which the Pb^2+^ and one Cu^+^ lie, and θ is in the perpendicular plane. (c) Rotational TCF for the MLWFC dipole moment of the Pb^2+^ lone pair.

Neutron diffraction confirms that the structure of aikinite (Figure 1) contains three crystallographically distinct cation sites, which are occupied, in a fully ordered fashion, by Bi^3+^, Pb^2+^, and Cu^+^ cations. The Bi^3+^ cation adopts a highly distorted octahedral coordination (Figure 3a) and forms one-dimensional [BiS_4_]^−^ chains of edge-sharing of [BiS_6_]^3–^ octahedra, oriented parallel to the *b*-axis (Figure 3b**)**. The highly distorted coordination of the [BiS_6_]^3–^ octahedra, with three shorter and three longer Bi–S distances, arises from the displacement of the central Bi^3+^ cation toward one of the octahedral faces. The marked polyhedral distortion is reflected in large values of the bond-angle variance^62^ as well as non-zero values of bond-length distortion^62^ (Supporting Information, Table S5). In the structurally related Bi_2_S_3_, the displacement of the cation from the ideal center of the octahedron has been attributed to the effect of the 6s^2^ lone pair.^63^ The short Bi–S distances, which vary between 2.662(2) and 2.759(2) Å, are comparable to the sum of the covalent radii^64^ for Bi and S (*ca.* 2.53 Å). By contrast, the three longer Bi–S distances, which range between 2.962(2) and 3.145(2) Å, are larger than the sum of ionic radii for Bi^3+^ and S^2–^, which is *ca*. 2.87 Å (r(Bi^3+^) = 1.03 Å and r(S^2–^) = 1.84 Å).^65^ This indicates that the bonding environment around the Bi^3+^ cation is heterogeneous, comprising both weaker and stronger bonds (see bond valence sums, Supporting Information Table S4). The AIMD simulations are in excellent agreement with the structural analysis: the Bi–S radial pair distribution function (RDF) obtained from *NVT* AIMD shows a first peak centered on the shorter bond lengths and a shoulder related to the longer Bi–S distances (Figure 3c); the asymmetry of this peak is consistent with a stereochemically active lone pair, which is evident in the electron localization function (ELF) (Figure 4). The magnitude of the Bader charges (*vide infra*) also indicates substantial deviations from a purely ionic picture of the bonding.

The Cu^+^ cations adopt a distorted tetrahedral coordination (Figure 3d), with corner-sharing [CuS_4_]^7–^ tetrahedra forming chains parallel to the *b*-axis (Figure 3e). The Cu–S distances vary between 2.320(2) and 2.419(2) Å, which is consistent with the computed Cu–S RDF (Figure 3f). The significant deviation of the Bader charges (*vide infra*) for Cu^+^ and S^2–^ from the formal oxidation states suggests a large degree of covalency for the Cu–S bonds. Each Cu^+^ cation is surrounded by three Pb^2+^ cations at a distance of *ca*. 3.3 Å, which is smaller than the sum of their van der Waals’ radii^66^ (*ca.* 3.8 Å) and suggests a possible interaction between the 6s^2^ lone pairs of Pb^2+^ and the Cu^+^ cations. Although longer than the Pb–Cu distances, there are also Cu–Bi distances of *ca.* 3.5 and 3.7 Å, which are below the sum of the van der Waals’ radii^66^ for Bi and Cu (*ca.* 3.9 Å). These cation–cation distances are also evident in the Cu–Bi and Cu–Pb RDFs (Supporting Information, Figure S3). The Pb^2+^ cation is coordinated to seven S^2–^ anions, forming a capped octahedron (Figure 3g). Each [PbS_7_]^12–^ capped octahedron shares faces with four other capped octahedra to form ribbons with stoichiometry [PbS_3_]^−^ oriented along the *b*-axis (Figure 3h). The Pb–S distances, which range from 2.885(2) to 3.274(2) Å, are comparable to the sum of the ionic radii, which is *ca.* 3.07 Å (r(Pb^2+^) = 1.23 Å),^65^ and in agreement with the AIMD RDF (Figure 3i). In addition, each Pb^2+^ cation has three neighboring Cu^+^ cations at approximately 3.3 Å (Movie S1).

Analysis of the ELF (Figure 4a), which can be used to determine if the bonding interactions involve shared electrons (e.g., covalent bonding) or unshared electrons (e.g., ionic bonding),^67^ reveals the presence of directional covalent bonds between Bi^3+^ and S^2–^, as well as that of a lone pair on the Bi^3+^ cations. By contrast, the distribution around the Pb^2+^ cations in the ELF contours is reasonably isotropic and spherical. This is consistent with weak electrostatic interactions between the Pb^2+^ cations and neighboring atoms, and suggests that Pb^2+^ could be considered to be in a quasi-liquid state. The isotropic ELF can arise from the dynamic behavior of the Pb^2+^ lone pair, which would give rise to rotational motion, as recently found in halide perovskites.^68^

The presence of different types of bonding (bond heterogeneity) has been identified as a characteristic feature that can lead to increased phonon scattering, resulting in low thermal conductivity.^69−71^ Moreover, in agreement with a previous single-crystal X-ray diffraction study,^60^ the atomic displacement parameters (*U*_iso_) for the Cu^+^ and Pb^2+^ cations are larger than those of Bi^3+^ and S^2–^ (Table 1), and the RDFs involving Cu^+^ and Pb^2+^ (Figures 3f,i and S3) exhibit marked peak broadening with increasing temperature. The effect of temperature has been investigated by examining the displacements of the cations with respect to their equilibrium positions (Supporting Information, Figure S4**)**. This shows a larger response of the Cu^+^ and Pb^2+^ cations to increases in temperature. A large atomic displacement parameter has been related to weak interatomic bonding and “rattling”-like vibrations,^19,72^ or to an underlying distortion at the local scale, arising from uncorrelated lone-pair stereochemical activity.^73^ For aikinite, analysis of the AIMD trajectories performed with Wannier functions provides clear evidence of a dynamical effect arising from rotation of the Pb^2+^ lone pair (Figures 4 and Supporting Information, Figure S5 plus Movie S2). This analysis suggests that the large atomic displacement parameters for Cu^+^ and Pb^2+^ arise from the incoherent rotation of the Pb^2+^ lone pair, which is accompanied by cooperative displacements of the Pb^2+^ cation toward one of the three neighboring Cu^+^ cations due to attractive electrostatic interaction between the lone pair and the Cu^+^ cation. From the statistical analysis of the AIMD trajectories, we determined the maxima of the angular amplitude for the Pb^2+^ lone pair MLWFC dipole moment; the values are ϕ_max_ = 360^°^ and θ_max_ = 134^°^. These angles are consistent with rotation of the lone pair between the three Cu^+^ cations.

### Electronic Structure and Transport Properties

**Figure 5:**
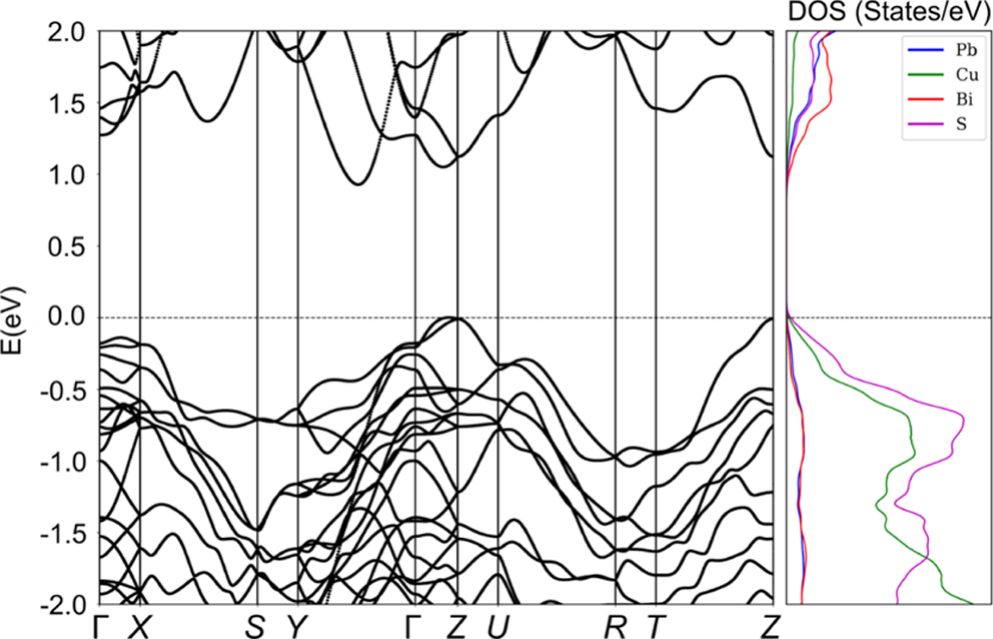
Electronic band structure (left panel) and atom-projected electron density of states (right panel) of aikinite. Spin–orbit coupling has been included in the calculation. Hubbard U corrections are included. The top of the valence band is set to 0 eV.

Aikinite is a semiconductor with a theoretical indirect band gap of 0.8 eV (Figure 5), which is in close agreement with the indirect band gap estimated from optical measurements, *ca*. 0.91 eV (Supporting Information, Figure S6). The computed partial density of states (Figure 5, right panel) shows that the major contribution at the top of the valence manifold arises, as expected, from the Cu^+^ and S^2–^ ions, which form corner-sharing tetrahedral chains. The top of the valence band has a multivalley character with large effective masses (Table S6) at the Γ, *X*, and *Z* high-symmetry points of the Brillouin zone (Figure 5, left panel). The effective masses, computed over the entire band structure (Table S6), are indicative of a high degree of anisotropy, which is not immediately evident from Figure 5, which shows the band structure along the high symmetry path of the Brillouin zone of aikinite. The dispersive valence band indicates significant deviation from ionic character, as demonstrated by the Bader charges, which are +1.3, +1.0, +0.5, and −1.0 for Bi, Pb, Cu, and S, respectively, at *T* = 0 K (interestingly, the values at *T* = 300 K are +1.5/+2.0, +1.0, +1.0, and −1.0/–1.5, respectively). The features of the band structure suggest a large Seebeck coefficient for *p*-type transport, arising from the large anisotropic effective masses at *Z*, and the high electrical resistivity.

**Figure 6:**
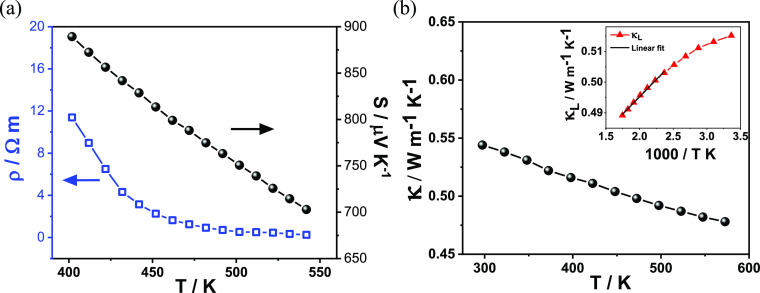
(a) Temperature dependence of the electrical resistivity (ρ) and the Seebeck coefficient (*S*) for aikinite, consistent with *p*-type semiconducting behavior. (b) Temperature dependence of the total thermal conductivity (κ) of aikinite. The inset shows a linear fit (black line) of the lattice thermal conductivity (κ_L_) plotted as a function of inverse temperature.

The conclusions from the band structure calculations are consistent with the experimentally determined Seebeck coefficient (*S*) (Figure 6a), which shows large, positive, and monotonically decreasing values between 423 and 573 K, indicating that aikinite is a nondegenerate *p*-type semiconductor. Measurements also indicate that CuPbBiS_3_ is highly resistive (1.27 × 10^3^ Ω cm at 423 K, Figure 6a), with a very low intrinsic charge carrier concentration, *ca*. 10^14^ cm^–3^, comparable to those determined for other undoped members of the aikinite-bismuthinite series (4.5 × 10^12^ cm^–3^ for CuPbBi_5_S_9_ and 3.7 × 10^16^ for Bi_2_S_3_).^29,31^ Unlike the intermediate composition CuPbBi_5_S_9_ (*x* = 2/3) in the bismuthinite-aikinite series Cu_1–*x*_□_*x*_Pb_1–*x*_Bi_1+*x*_S_3_,^31^ which exhibits *n*-type conductivity probably because of sulfur vacancies, aikinite CuPbBiS_3_ (*x* = 0), as prepared following the synthesis route described herein, retains *p*-type conductivity even after repeated heated-cooling cycles (Supporting Information, Figure S7). TGA measurements (Supporting Information, Figure S8) confirm the stability of this material, which is thermally stable up to 800 K under an inert atmosphere, while DSC data are consistent with the absence of phase transitions (Supporting Information, Figure S9). While in aikinite, there is a continuous one-dimensional network of corner-sharing [CuS_4_]^7–^ tetrahedra, which facilitates *p*-type electrical conduction, other members of the bismuthinite-aikinite series, Cu_1–*x*_□_*x*_Pb_1–*x*_Bi_1+*x*_S_3_, with *x* > 0 contain vacant sites in the one-dimensional [Cu_1-*x*_□_*x*_S_3_]^(5+*x*)–^ chains instead of a continuous network. Therefore, in copper-deficient materials, such as CuPbBi_5_S_9_, *n*-type electrical conduction involves the Bi–S network, as previously discussed by Maji *et al.*(31)

A study of the formation of defects in Bi_2_S_3_ (*x* = 1) has shown that for the S rich limit, Bi vacancies (□_Bi_^‴^) and S antisite defects (S_Bi_^⁗^) are dominant, while in the Bi rich limit, the dominant defects are S vacancies (□_S_^••^) and Bi antisite defects (Bi_S_^•••••^).^74^ The situation for CuPbBiS_3_ is more complex. Acceptor defects that can occur in aikinite include copper vacancies (□_Cu_^′^) usually observed in chalcogenides, lead vacancies (□_Pb_^″^), antisite defects of Cu at the Pb site (Cu_Pb_^′^) or Pb on the Bi site (). In addition, bismuth vacancies (□_Bi_^‴^), owing to the volatility of bismuth, could also form, although under the synthesis conditions (low temperature annealing and hot-pressing) used here, this is considered unlikely. A detailed study of the energetics of defect formation is ongoing.

### Lattice Thermal Conductivity and Elastic and Vibrational Properties

The total thermal conductivity of CuPbBiS_3_ (Figure 6b) is extremely low (∼0.5 W m^–1^ K^–1^) and is dominated by the lattice component, κ_L_ (Supporting Information, Figure S10a), which constitutes approximately 99% of the total thermal conductivity. κ_L_ follows a T^–1^ law only at temperatures above 450 K, indicating a significant contribution from Umklapp type scattering above this temperature (Figure 6b, **inset**). Despite the differences in cation ordering between CuPbBi_5_S_9_, in which there is disorder between Cu^+^ and vacancies on the copper site and between Pb^2+^ and Bi^3+^ cations on the M1 site,^31^ and CuPbBiS_3_, in which Cu^+^, Pb^2+^, and Bi^3+^ cations are fully ordered, the thermal conductivities of CuPbBiS_3_ and CuPbBi_5_S_9_ are very similar. By contrast, Bi_2_S_3_ (*x* = 1) exhibits significantly larger thermal conductivities; for highly oriented ingots, the thermal conductivity is ∼1.3 W m^–1^ K^–1^ along the *b*-axis and ∼0.9 W m^–1^ K^–1^ along *a* and *c*,^29^ while polycrystalline Bi_2_S_3_ exhibits a total thermal conductivity of ∼0.87 W m^–1^ K^–1^ at room temperature.^74^ Therefore, the reduction in thermal conductivity that occurs in Cu_1–*x*_□_*x*_Pb_1–*x*_Bi_1+*x*_S_3_ for *x* < 1 suggests that Cu^+^ and Pb^2+^ cations play a key role in the heat transport. To investigate the origin of the ultralow thermal conductivity in aikinite, sound velocity measurements were performed, from which elastic properties were derived (Table S7). The sound velocities obtained computationally are in good agreement with the experimental values (Table 2**)**. Both the transverse (1560 m s^–1^) and the longitudinal sound velocities (2771 m s^–1^), which can be related to the group velocities of the heat-carrying acoustic phonons, are low and comparable to those for CuPbBi_5_S_9_ (*x* = 2/3).^31,32^ Since it has been shown^75^ that, above the Debye temperature, κ_L_ is directly proportional to the cube of the average sound velocity (, where *A* is a proportionality constant), a low sound velocity is expected to result in low thermal conductivity. For aikinite, the minimum value of κ_L_, according to the Cahill-Watson-Pohl (CWP) model,^37^ which describes the limit for amorphous and strongly disordered materials, is *ca*. 0.41 W m^–1^ K^–1^. This is similar to the experimental value of 0.48 at 573 K determined here. The minimum κ_L_ can also be calculated by considering a diffusive mechanism in which nonpropagating (i.e., not phononic) atomic vibrations, known as diffusons, carry heat by diffusion.^76^ This leads to an estimate of the diffuson thermal conductivity, κ_diff_ ∼ 0.26 W m^–1^ K^–1^. This represents the limit for entirely diffusive mediated transport and is significantly lower than the values found for aikinite, providing a strong indicator that phonons contribute to heat transport in aikinite. Taking into account that κ_L_ = 1/3*C*_p_*v*_a_*l* (where *C*_p_ is the heat capacity per unit volume, and *l* is the phonon mean-free-path), the estimated phonon mean-free-path is *ca*. 5 Å, which is approximately twice the interatomic spacing in aikinite and comparable to the *b* lattice parameter.

**Table 2:** Experimentally and Computationally Determined Sound Velocities and Elastic Properties Derived from the Sound Velocities for Aikinite

	sound velocity (m s^–1^)			derived parameters			
	transverse *v*_T_	longitudinal *v*_L_	average sound velocity *v*_a_	Poisson’s ratio	Young’s modulus (GPa)	Grüneisen parameter	Debye temperature θ_D_ (K)
Exp	1560	2771	1736	0.27	42.7	1.59	183
Th (phonon)^a^	1599	3123	1929	0.28		1.64	
	1865						
Th (elastic)^b^	1733	3199	1933	0.29		1.73	

a Determined using the phonon dispersion.

b Determined using the elastic constants.

The Grüneisen parameter derived from the sound velocity measurements is large, γ ∼ 1.59, indicating a high degree of anharmonicity and comparable to values found for other thermoelectric materials with low thermal conductivities (Supporting Information, Table S8**)**. The Grüneisen parameter obtained here is similar to that reported for other members of the Cu_1–*x*_□_*x*_Pb_1–*x*_Bi_1+*x*_S_3_ series (Supporting Information, Table S9). Anharmonicity enhances phonon–phonon scattering processes, hence lowering the lattice thermal conductivity. The Young’s modulus (*E*), extracted from the sound velocities (Table 2), is rather low (Supporting Information, Table S8), and similar to values reported for other members of the Cu_1–*x*_□_*x*_Pb_1–*x*_Bi_1+*x*_S_3_ series (Supporting Information, Table S9). As the Young’s modulus is related to the stiffness of the atomic bonds, this is indicative of weaker interatomic bonding. This supports the conclusions drawn from the structural analysis.

**Figure 7:**
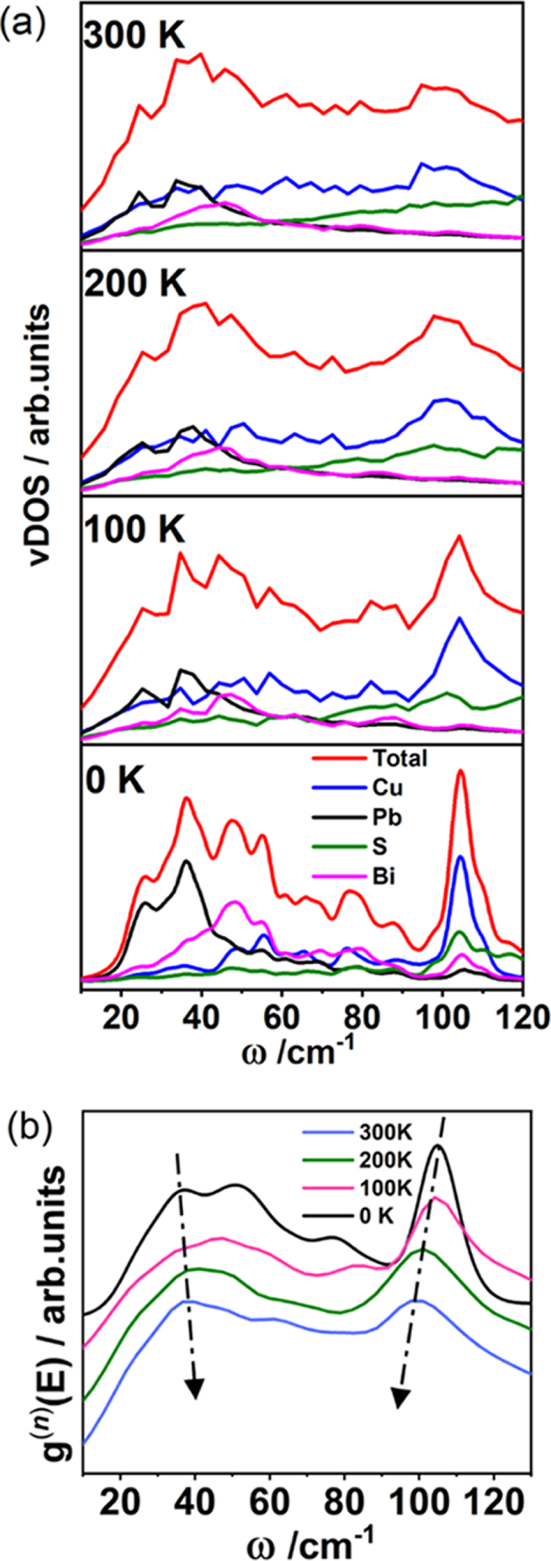
(a) Total (red line) and atom-resolved vDOS of aikinite at different temperatures. Blue, black, green, and purple lines correspond to Cu^+^, Pb^2+^, S^2–^, and Bi^3+^ ions, respectively. The vDOS at 0 K was computed using Quantum ESPRESSO and those between 100 and 300 K by AIMD. (b) Neutron-weighted total vDOS calculated with the LET instrumental resolution function as a function of temperature. Arrows indicate shifts in peak positions.

Figure 7 presents the atom-resolved phonon density of states (vDOS) computed at 100, 200, and 300 K by AIMD, compared with the vDOS at 0 K^31^ calculated using Quantum ESPRESSO. These results should also be compared with the dispersion curves presented in ref (31), which show optical modes with very low frequencies (∼20–50 cm^–1^). It has been shown that a low cutoff frequency of acoustic phonons, which can be ascribed to weak bonding and a correspondingly low sound velocity, is a good indicator for low thermal conductivity.^77^ Moreover, the presence of low-frequency optical modes close to the acoustic mode frequencies, as is the case here, can lead to phonon scattering and affect thermal transport.

Low-frequency optical modes in the range 20–50 cm^–1^ and Einstein-like modes centered around 100 cm^–1^ are evident in the vDOS of aikinite (Figure 7). The atom-resolved vDOS shows that the main contribution at low frequencies is from Pb^2+^, with a smaller contribution from Bi^3+^. It is interesting to note that the difference between the Bi^3+^ and Pb^2+^ modes at low frequencies cannot be accounted for in terms of the variation in atomic mass (208.9 and 207.2 amu, respectively), but is consistent with the weak bonding of Pb^2+^ highlighted by the structural analysis. In addition, analysis of the vDOS computed by AIMD as a function of temperature (Figure 7a) indicates that, with increasing temperature, the Cu^+^ vibrational modes spread and shift to lower frequencies, overlapping more with phonons with a large Pb^2+^ component, while the Bi^3+^ contribution remains largely unchanged (Figure 7a). The shift, with increasing temperature, of the Cu^+^ vibrational modes to lower frequencies and of the peak at *ca.* 31 cm^–1^ toward higher frequencies is also evident in the neutron*-*weighted total calculated vDOS convolved with the LET instrumental resolution function (Figure 7b).

**Figure 8:**
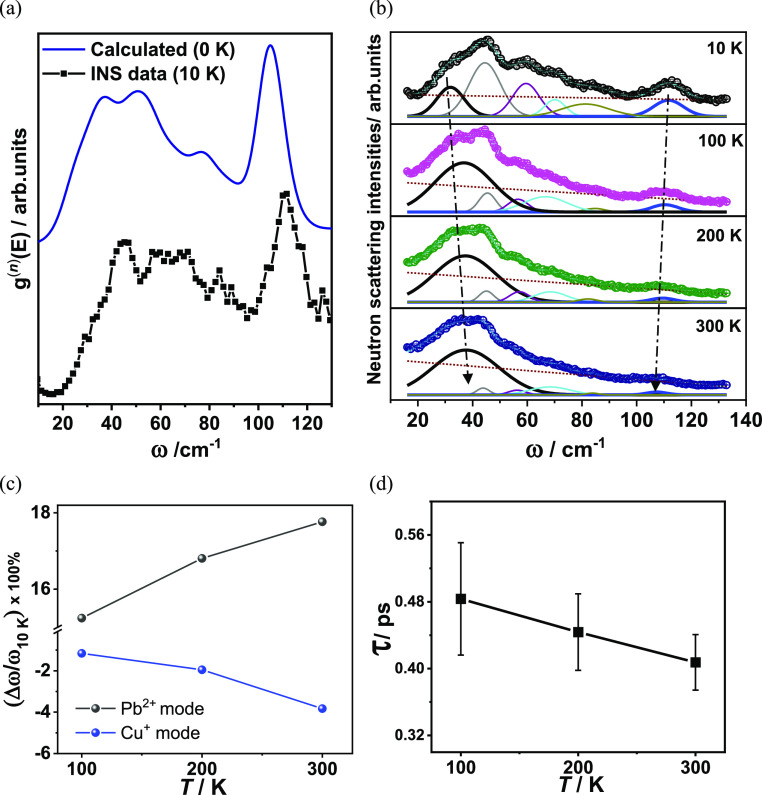
(a) Calculated neutron-weighted total vDOS (g^n^(E)) (convolved with the instrumental resolution) at 0 K (blue line) and experimental neutron-weighted vDOS (black) at 10 K. Data have been normalized. (b) Neutron scattering intensities as a function of temperature. Gaussian fits for the six peaks are shown. The Gaussian corresponding to the Pb^2+^-based vibration is shown as a black line, and that for the Cu^+^ rattling vibration is shown as a blue line. Experimental data have been offset along the *y* axis with respect to the Gaussian fits for clarity. The red dotted line shows the fitted background. Arrows highlight the change in peak position for the Pb^2+^-based and Cu^+^ rattling vibrations with temperature. (c) Percentage change in the energy of the phonon mode between 10 K and a temperature *T*. The gray line corresponds to the Pb^2+^ mode (ω = 32 cm^–1^) and the blue line corresponds to the Cu^+^ mode (ω = 110 cm^–1^). (d) Temperature dependence of the phonon lifetime of the Pb^2+^ mode.

**Figure 9:**
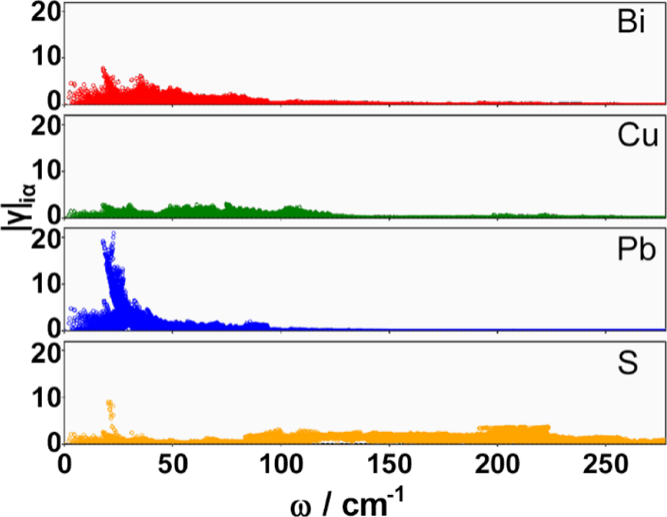
Contribution of each atomic species to the total Grüneisen parameter as a function of the mode frequency. Red, green, blue, and yellow points correspond to Bi^3+^, Cu^+^, Pb^2+^, and S^2–^ contributions, respectively.

In order to confirm, experimentally, the presence of the low-energy phonon modes of Pb^2+^ and the Einstein-like modes arising from the Cu^+^ vibrations described above, temperature-dependent INS data were collected. The calculated neutron-weighted vDOS (convolved with a Gaussian function approximating the instrumental resolution) and the experimental vDOS are in very good agreement (Figure 8a and Supporting Information, Figure S11). The slight shift in frequencies between the experimental and calculated vDOS is due to the PBE functional used in the calculations, which is known to underestimate bond strengths, and the slight differences in peak intensities are related to approximations made when convolving the INS instrumental resolution with the calculated vDOS. A comparison with the partial calculated vDOS (Figure 7a) allows us to assign the first peak in the experimental vDOS, which at 10 K is centered at 31.7 cm^–1^, to primarily Pb^2+^-based vibrations. Of the other five peaks present in the INS data, most have contributions from multiple atoms, and only that at 110.7 cm^–1^ can be assigned to the Einstein-like modes of Cu^+^. Examination of the experimental vDOS collected as a function of temperature (Figure 8b) shows that peaks shift significantly with temperature (Table S10). In particular, with increasing temperature (Figure 8c), the Cu^+^ mode softens, in agreement with the findings of the computed vDOS. By contrast, the phonon peak corresponding to the Pb^2+^-based mode shifts to a higher frequency (Figure 8c). Such “hardening” of the Pb^2+^ mode with increasing temperature is indicative of anharmonic behavior and is therefore likely to be a key contributor to the ultralow thermal conductivity of aikinite. To evaluate the role of Pb^2+^ in lowering the lattice thermal conductivity, we used the quasi-harmonic approximation to estimate the degree of anharmonicity through the mode-resolved Grüneisen parameter, γ_iα_, and its atom projection as a function of the frequency (Figure 9). This analysis provides clear evidence for significant anharmonicity. While in aikinite, anharmonicity could be associated with the presence of the 6s^2^ lone pair of electrons found in Pb^2+^ and Bi^3+^ cations, examination of Figure 9 reveals that at low frequencies, over the range 20–50 cm^–1^, the magnitude of mode-resolved Grüneisen parameter is significantly higher for Pb^2+^ than for the other atoms. Finally, the rotational TCF (Figure 4c) associated with the Pb^2+^ lone pair shows a rotational time scale of ∼80 fs; this corresponds to a frequency of ∼66 cm^–1^, comparable with the low-frequency mode of Cu^+^. This observation is consistent with the cooperative interaction between the rotation of the Pb^2+^ lone pair and the Cu^+^ cations discussed earlier.

Other members of the bismuthinite-aikinite series, Cu_1–*x*_□_*x*_Pb_1–*x*_Bi_1+*x*_S_3_, are isostructural with aikinite (*x* = 0), and when *x* < 1, also contain Cu^+^ and Pb^2+^ cations at relatively short distances, which could facilitate coupling of rotating lone pairs and the vibrational motion of the Cu^+^ cations. Moreover, based on the limited data available in the literature, members of this series with *x* < 1 exhibit similarly low values of thermal conductivity to those of aikinite. This ultralow thermal conductivity has previously been ascribed to the complex crystal structure with a large unit cell and heavy atoms,^32^ or to disorder.^78^ However, given that aikinite has an ultralow thermal conductivity even with complete ordering of the Cu^+^, Pb^2+^, and Bi^3+^ cations, we can effectively discount disorder as the origin of the ultralow thermal conductivity in other members of the Cu_1–*x*_□_*x*_Pb_1–*x*_Bi_1+*x*_S_3_ series with *x* < 1. Furthermore, the larger thermal conductivity of Bi_2_S_3_ (*x* = 1) when compared to other members of the series indicates that the Cu^+^ and Pb^2+^ cations play a key role in heat transport. Together, these observations suggest that the mechanism of lone pair rotation we have unveiled in aikinite, which we have demonstrated is a key contributor to the scattering effects that lower the thermal conductivity, is also at play in other members of the bismuthinite-aikinite series, Cu_1–*x*_□_*x*_Pb_1–*x*_Bi_1+*x*_S_3_.

It is extremely challenging to extract lifetimes from INS data collected on a powder, owing to the averaging over Brillouin zones that occurs in such an experiment.^69^ This is especially true when, with increasing temperature, modes are shifting very significantly in frequency and in different directions, with corresponding changes to the underlying dispersion. As a result, peaks might sharpen or broaden independent of broadening from phonon–phonon scattering. In the INS data for aikinite, the full width at half maxima (FWHM) of the majority of the peaks decreases with increasing temperature (Supporting Information Table S11), indicating that the modes sharpen at higher temperatures. Such a sharpening could be related to a flattening of the dispersion and, thereby, a reduction in the optical mode group velocities. However, for Pb^2+^, the low-energy mode broadens with increasing temperature (Table 3 and Supporting Information Table S11). While it is not possible to disentangle the likely changes to the dispersion from phonon scattering, if we assume that the broadening is all from phonon–phonon scattering, we can extract a rather low lifetime of 0.41(3) ps at 300 K (Table 3 and Figure 8d). This compares favorably with the values of ∼3 ps, ∼0.66 ps and ∼2 ps for PbTe,^79^ TlInTe_2_^69^ or Na_0.8_CoO_2_,^80^ respectively. While the lifetime determined here should be considered a lower bound, since it is likely that at least some of the broadening is due to changes in the dispersion, this lifetime would correspond to a phonon mean-free-path (*l* = *v*_a_ τ) of *ca.* 7 Å, which is in reasonable agreement with the estimated mean-free-path based on the lattice thermal conductivity.

**Table 3:** Phonon Lifetime (τ) for the Pb^2+^ Mode^a^

*T* (K)	peak center (cm^–1^)	FWHM (cm^–1^)	Δ_FWHM_ (cm^–1^)	τ (ps)
10	31.7(4)	11.6(5)		
100	36.5(4)	24.7(8)	21.7(4)	0.48(7)
200	36.9(3)	26.4(5)	23.7(3)	0.44(5)
300	37.3(2)	28.3(4)	25.8(3)	0.41(3)

a Calculated using the relation^69,81^
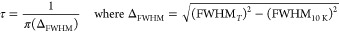

## Conclusions

In aikinite, the combination of bond heterogeneity and the presence of heavy-metal cations with stereochemically-active lone pairs leads to an exceptionally low thermal conductivity. Moreover, the Cu^+^ and Pb^2+^ cations have large atomic displacement parameters and contribute to the same low-frequency vibrational manifold. With increasing temperature, the Cu^+^ contributions in the low-frequency region increase. This is consistent with a thermally activated interaction between Cu^+^ and Pb^2+^ cations. Using *ab-initio* molecular dynamics and Wannier function analysis, we have characterized the weak and isotropic bonding of the Pb^2+^ cations that facilitates the rotation of the 6s^2^ lone pair. In turn, such rotations influence the Cu^+^ dynamics and reduce the thermal conductivity. The interaction mechanism can be rationalized by considering the electrostatic attraction between the thermally activated lone pairs on the Pb^2+^ cations, which are rotating, and the Cu^+^ cations. Given that synthetic samples in the bismuthinite-aikinite series, Cu_1–*x*_□_*x*_Pb_1–*x*_Bi_1+*x*_S_3_, are isostructural with aikinite (*x* = 0), it is highly likely that the ultralow thermal conductivity observed for this family of materials is also a consequence of the cooperative interaction between the rotating lone pair on the Pb^2+^ cations and the Cu^+^ cations.

Lone pair rotation in solids is an emergent phenomenon, recently reported in halide perovskites, although it has been suggested that such rotational motion may also occur in other solids containing lone-pair electrons.^57^ The work presented here provides clear evidence for the dynamical behavior of the Pb^2+^ lone pair and its contribution to lowering thermal conductivity, in a family of thermoelectric sulfides, Cu_1–*x*_□_*x*_Pb_1–*x*_Bi_1+*x*_S_3_. We have demonstrated, for the first time, that the coupling of rotating lone pairs with vibrational motion is an effective mechanism to achieve ultralow thermal conductivity in crystalline materials. We suggest that this mechanism may also occur in other families of materials, including halide perovskites such as CsSnBr_3-x_I_*x*_, which also exhibit ultralow thermal conductivities.^82^ Therefore, the results presented here offer new insights for the search for materials with ultralow thermal conductivity.

## Supporting Information Available

Details of the methodology, Helmholtz free energy, internal energy, entropy and specific heat, Rietveld refinements using different models, bond distances, bond valence sums and distortion parameters, RDF plots, ion displacement plots, diffuse reflectance, TGA, DSC, heat capacity, electrical and thermal transport data, calculated effective masses, computed vibrational DOS determined using INS data and derived parameters, and computed elastic constants (PDF)
Vibrations of Pb^2+^ (black sphere) and Cu^+^ (blue sphere), with distances shown (MP4)
AIMD showing the Pb^2+^ lone pair rotation (MP4)
