# Supplementary material for: Lone Pair Rotation and Bond Heterogeneity Leading to Ultralow Thermal Conductivity in Aikinite
Source: J Am Chem Soc. 2023 Apr 13;145(16):9313–25. doi: 10.1021/jacs.3c02536 (PMC10141412; doi:10.1021/jacs.3c02536)
Supplement: Supplementary file 1 — ja3c02536_si_001.pdf [file ja3c02536_si_001.pdf]

Supporting Information for

Lone pair rotation and bond heterogeneity leading to ultralow thermal conductivity in aikinite

Virginia Carnevali,[1†§] Shriparna Mukherjee,[2§] David J. Voneshen,[3,4] Krishnendu Maji,[5] Emmanuel Guilmeau,[5] Anthony V. Powell,[2] Paz Vaqueiro,[2*] Marco Fornari[1*]

[1] Department of Physics and Science of Advanced Materials Program, Central Michigan University, Mt. Pleasant, MI 48859, USA

[2]Department of Chemistry, University of Reading, Whiteknights, Reading, RG6 6DX, United Kingdom.

[3]ISIS Pulsed Neutron and Muon Source, Rutherford Appleton Laboratory, Chilton, Didcot, Oxon OX11 0QX, United Kingdom

[4]Department of Physics, Royal Holloway University of London, Egham, TW20 0EX, United Kingdom

[5]CRISMAT, CNRS, Normandie Univ, ENSICAEN, UNICAEN, 14000 Caen, France

†Present address: Laboratory of Computational Chemistry and Biochemistry, École Polytechnique Fédérale de Lausanne, 1015 Lausanne, Switzerland

§ These authors contributed equally to this work.

* Corresponding authors: E-mail addresses: p.vaqueiro@reading.ac.uk (Paz Vaqueiro), marco.fornari@cmich.edu (Marco Fornari)

Methods

Polyhedral distortions: The bond angle variance (σ^2) is calculated using the relation, $\sigma^2 = \frac{1}{m-1}\sum_{i=1}^{m}(\theta_i - \theta_{poly})^2$, the bond length distortion (distortion index) as defined by Baur[1] as, $D = \frac{1}{n}\sum_{i=1}^{n}\frac{|l_i - l_{av}|}{l_{av}}$ and the quadratic elongation as $<\lambda> = \frac{1}{n}\sum_{i=1}^{n}\left(\frac{l_i}{l_{poly}}\right)$, which gives a quantitative measure of polyhedral distortion.

The subscript "poly" refers to the polyhedron type (octahedron or tetrahedron), with angles θ_{poly} as 90° and 109.47° respectively. θ_i refers to the *i*th bond angle; the summation is taken over the *m* angles of the polyhedron or the *n* vertexes of the polyhedron, l_i is the distance from the central atom to the *i*th coordinating atom, l_{poly} is the centre-to-vertex distance of a regular polyhedron of same volume, and l_{av} is the average bond length. θ_{poly} in the bond angle variance and l_{poly} in the quadratic elongation calculation could be defined only for a regular polyhedron and hence could not be calculated for the PbS_7 capped octahedron. For an undistorted polyhedron, the bond angle variance and distortion index would be 0 whereas the quadratic elongation would be 1.

The number of atoms coordinated to a central atom in a coordination polyhedron is defined as the effective coordination number (ECoN) and is ideally 4, 6 and 7 for a tetrahedron, octahedron and capped octahedron respectively. ECoN was obtained using the formulation of Robinson *et.al.*[2] and Hoppe[3] as implemented in VESTA where the surrounding atoms to a central atom in a coordination polyhedron are given a weighting scheme with numbers between 0 and 1; as the distance from the central atom to a surrounding atom increases, this number gets closer to zero.

Sound velocity measurements: The following expressions were used to extract elastic parameters, the Debye temperature and the Grüneisen parameter from the sound velocity measurements.

Average velocity

$$v_a = \left(\frac{1}{3}\left[\frac{1}{v_l^3} + \frac{2}{v_t^3}\right]\right)^{-1/3}$$

Poisson ratio

$$v_p = \frac{1 - 2(v_t/v_l)^2}{2 - 2(v_t/v_l)^2}$$

Grüneisen parameter

$$\gamma = \frac{3}{2}\left(\frac{1+v_p}{2-3v_p}\right)$$

Young's modulus

$$E = \frac{\rho v_l^2(1+v_p)(1-2v_p)}{(1-v_p)}$$

Debye temperature

$$\theta_D = \frac{h}{k_B}\left(\frac{3N}{4\pi V}\right)^{-1/3} v_a$$

Where N is the number of atoms in the unit cell (N = 24), V is the volume of the unit cell as obtained from refinement (534.250(4) Å^3), and ρ is the Archimedes' density 6.921 g cm^{-3};

Vibrational properties: The calculations of the transverse and longitudinal sound velocities have been derived using two different approaches. In the first method, the first three phonon branches have been computed on a set of three uniform spherical grids with increasing radius centered in Γ and then averaging the angular coefficient of the linear fits computed in each direction of the sphere. In the second method, the elastic constants are used to compute the bulk (B) and shear (G) moduli, using the Voigt approximation

$$B = \frac{1}{9}[(C_{11}+C_{22}+C_{33})+2(C_{12}+C_{23}+C_{13})]$$

$$G = \frac{1}{15}[(C_{11}+C_{22}+C_{33})-(C_{12}+C_{23}+C_{13})]+\frac{1}{5}(C_{44}+C_{55}+C_{66})\,.$$

The longitudinal and transverse sound velocities are then derived as

$$v_L = \left[\frac{1}{\rho}\left(B+\frac{4}{3}G\right)\right]^{\frac{1}{2}} \qquad v_T = \left(\frac{G}{\rho}\right)^{\frac{1}{2}},$$

where ρ is the mass density of the material. The average sound velocity reads as

$$\bar{v} = \left[\frac{1}{3}\left(\frac{2}{v_T^3}+\frac{1}{v_L^3}\right)\right]^{-\frac{1}{3}}.$$

Within the quasi-harmonic approximation, we compute the mode resolved Grüneisen parameter γ_{q_j} for the wave vector q and the phonon branch j and its contribution by each atomic species by taking the derivative of the dynamical matrix with respect to the volume as explained

by Siloi *et al.*[4]. In order to have a complete description of the system's equilibrium, thermodynamical potential functions such as Helmholtz free energy *F*, internal energy *E*, entropy *S* and specific heat C_v at zero pressure are obtained using the calculated phonon density of states employing the quasi-harmonic approximation. The following equations have been used to calculate *F; E; S;* and C_v:[5,6]

$$F = 3nNk_BT\int_0^{\omega_{max}} \ln\left(2\,sinh\left(\frac{\hbar\omega}{2k_BT}\right)\right) g(\omega)d\omega$$

$$E = 3nN\frac{\hbar}{2}\int_0^{\omega_{max}} \omega\coth\left(\frac{\hbar\omega}{2k_BT}\right) g(\omega)d\omega$$

$$S = 3nNk_B\int_0^{\omega_{max}} \left[\frac{\hbar\omega}{2k_BT}\coth\left(\frac{\hbar\omega}{2k_BT}\right) - \ln\left(2\sinh\left(\frac{\hbar\omega}{2k_BT}\right)\right)\right] g(\omega)d\omega$$

$$C_v = 3nNk_B\int_0^{\omega_{max}} \left(\frac{\hbar\omega}{2k_BT}\right)^2 \mathrm{csch}^2\left(\frac{\hbar\omega}{2k_BT}\right) g(\omega)d\omega$$

where k_B is the Boltzmann constant, *n* is the number of atoms per cell, *N* is the number of cells, ω is the phonon frequency, ω_{max} is the cut-off phonon frequency, and $g(\omega)$ is the normalized phonon density of states.

Calculation of the minimum thermal conductivity:

Considering the Cahill-Watson-Pohl (CWP) model, where the transport of thermal energy within a material takes place via a random walk to the nearest neighbour of a localized Einstein oscillator, the minimum thermal conductivity at high temperature can be approximated as,

$$\kappa_{min,CWP} \approx 1.21n^{2/3}k_B\frac{1}{3}(v_L + 2v_T)$$

Using this relation, the minimum thermal conductivity is ca. 0.414 W m^{-1} K^{-1}.

Based on the Allen-Feldman theory,[7,8] the minimum thermal conductivity (diffusive thermal conductivity) at high temperature can be approximated as,

$$\kappa_{diff} \approx 0.76n^{2/3}k_B\frac{1}{3}(v_L + 2v_T)$$

Using this relation, the minimum thermal conductivity is ca. 0.260 W m^{-1} K^{-1}.

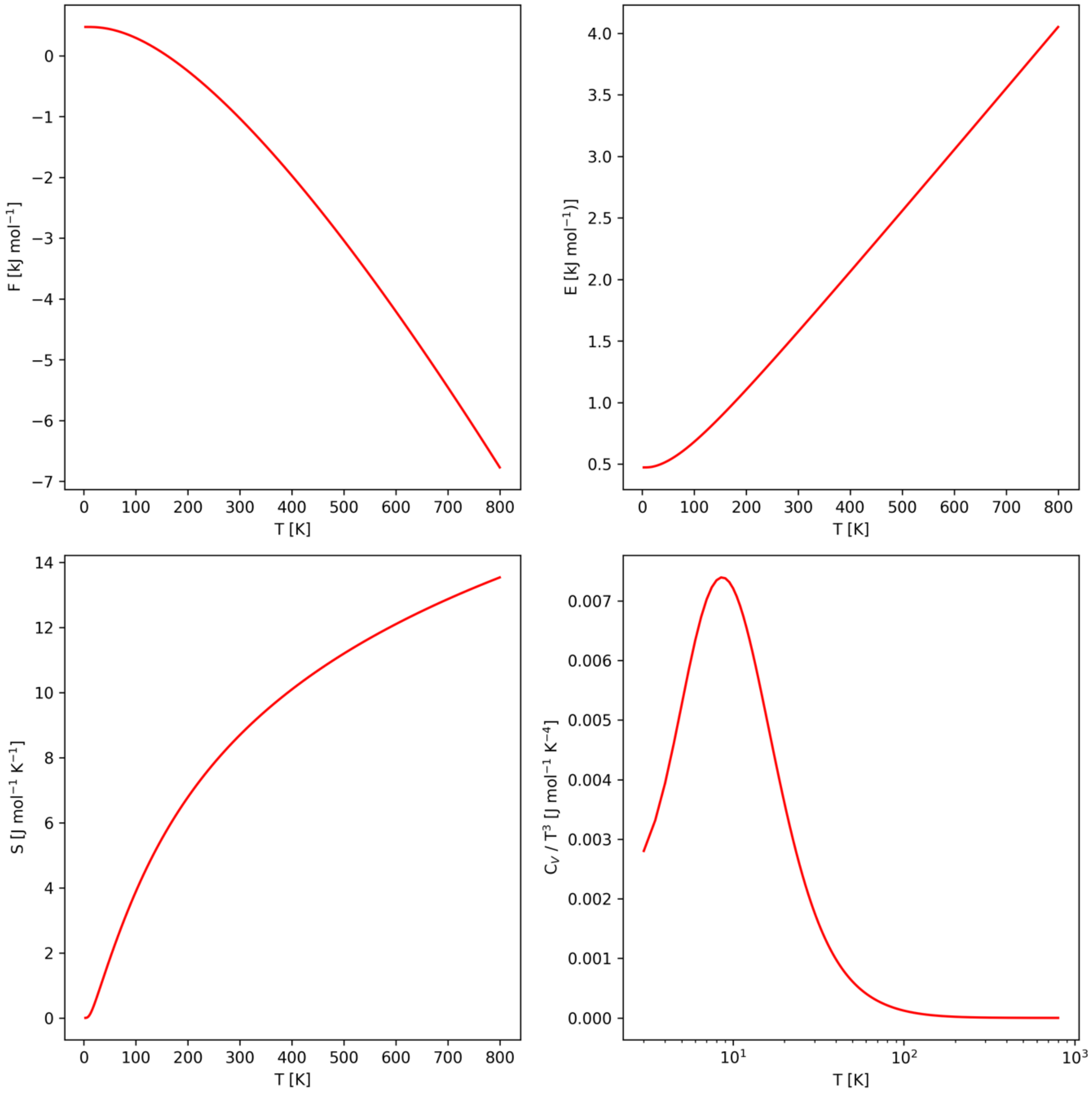

Figure S1. Computed Helmholtz free energy *F*, internal energy *E*, entropy *S* and specific heat C_v at zero pressure.

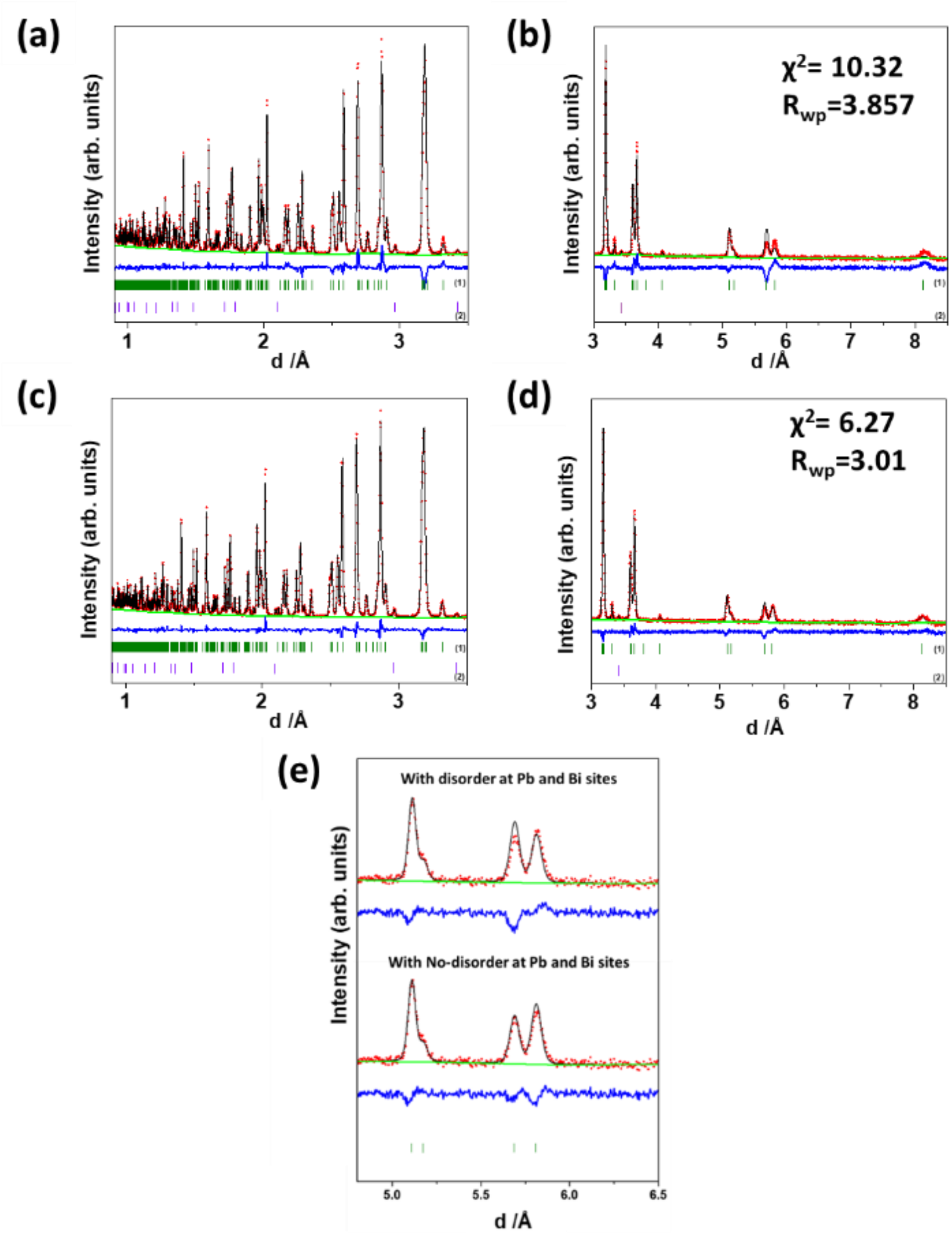

Figure S2. Rietveld refinement using neutron diffraction data for $CuPbBiS_3$. In (a) and (b), a structural model in which all Pb^{2+} cations are located on the M2 site and all Bi^{3+} cations on the M1 site (model (2)), has been used. In (c) and (d), a structural model in which Pb^{2+} and Bi^{3+} cations are disordered between the M1 and M2 sites (model (3)), has been used. (e) Comparison between the model with disorder at Pb and Bi sites and model (1), in which all Pb^{2+} cations are located on the M1 site and all Bi^{3+} cations on the M2 site, at *d*-spacings over the range $4.8 \leq d/Å \leq 6.5$; the top panel shows a clear mismatch between the observed and calculated neutron diffraction patterns for the model with disorder. Green ticks correspond to the reflection markers for the $CuPbBiS_3$ phase while the purple ticks correspond to those of PbS.

Refined parameters from Rietveld refinement of powder neutron diffraction:

Table S1. Distances(/Å) for Lead

Pb	Cu	3.385(1)		Pb	S1	2.989(2) ×2
Pb	Cu	3.3091(9)×2		Pb	S1	2.885(2)
				Pb	S2	3.274(2) ×2
				Pb	S3	2.993(2) ×2

Table S2. Distances(/Å) for Copper

Cu	Pb	3.385(1)		Cu	S1	2.320(2)
Cu	Pb	3.3091(9)×2		Cu	S2	2.419(2)
				Cu	S3	2.354(1) ×2

Table S3. Distances(/Å) for Bismuth

Bi	S1	2.962(2) ×2
Bi	S2	2.759(2) ×2
Bi	S2	3.145(2)
Bi	S3	2.662(2)

Table S4. Bond valence sums for Pb, Cu and Bi atoms

		d/Å	v	
Pb	S1	2.989(2)	0.297	×2
	S1	2.885(2)	0.394	
	S2	3.274(2)	0.137	×2
	S3	2.993(2)	0.294	×2
			1.861	

		d/Å	v	
Cu	S1	2.320(2)	0.319	
	S2	2.419(2)	0.244	
	S3	2.354(1)	0.292	×2
			1.147	

		d/Å	v	
Bi	S1	2.962(2)	0.347	×2
	S2	2.759(2)	0.600	×2
	S2	3.145(2)	0.211	
	S3	2.662(2)	0.779	
			2.884	

Table S5. Polyhedral distortions for the CuS_4, BiS_6 and PbS_7 polyhedra calculated as described in refs.1, 2 and 3.

Polyhedra	Bond angle variance(deg.)	Bond length distortion (D)	Quadratic elongation (λ)	Effective coordination number
CuS_4	73.39	0.012	1.02	3.97
BiS_6	190.20	0.052	1.07	5.15
PbS_7	-	0.041	-	6.39

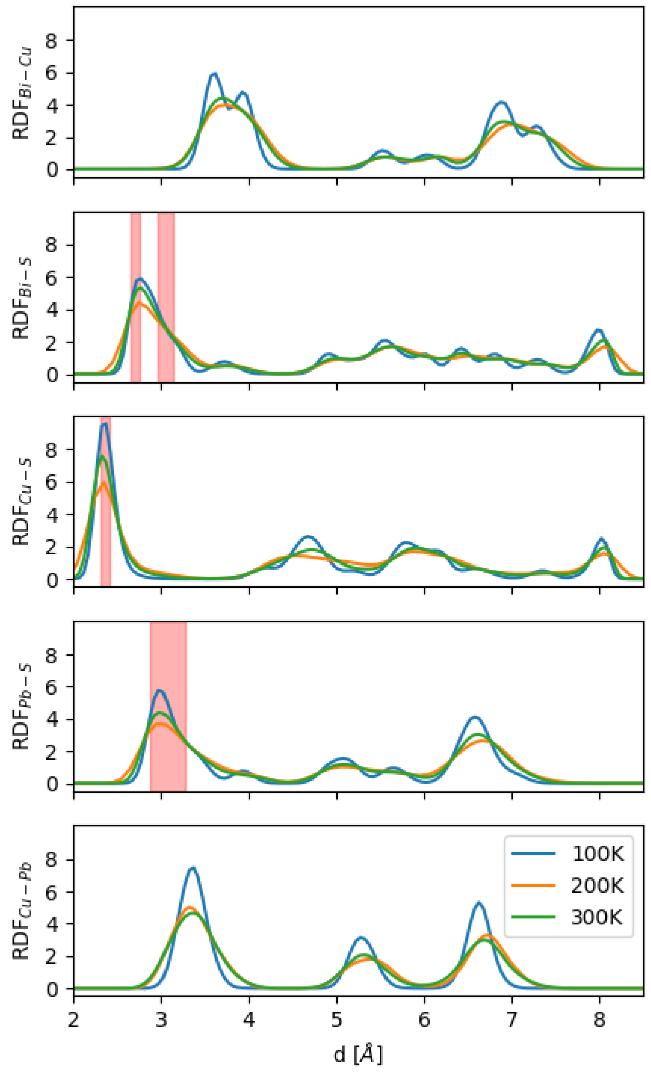

Figure S3. RDF computed from NVT *ab initio* MD trajectory: blue, orange, green line corresponds to simulations at 100 , 200 and 300 K respectively. The red shadow area highlights the distances determined experimentally from Rietveld analysis of powder neutron diffraction data.

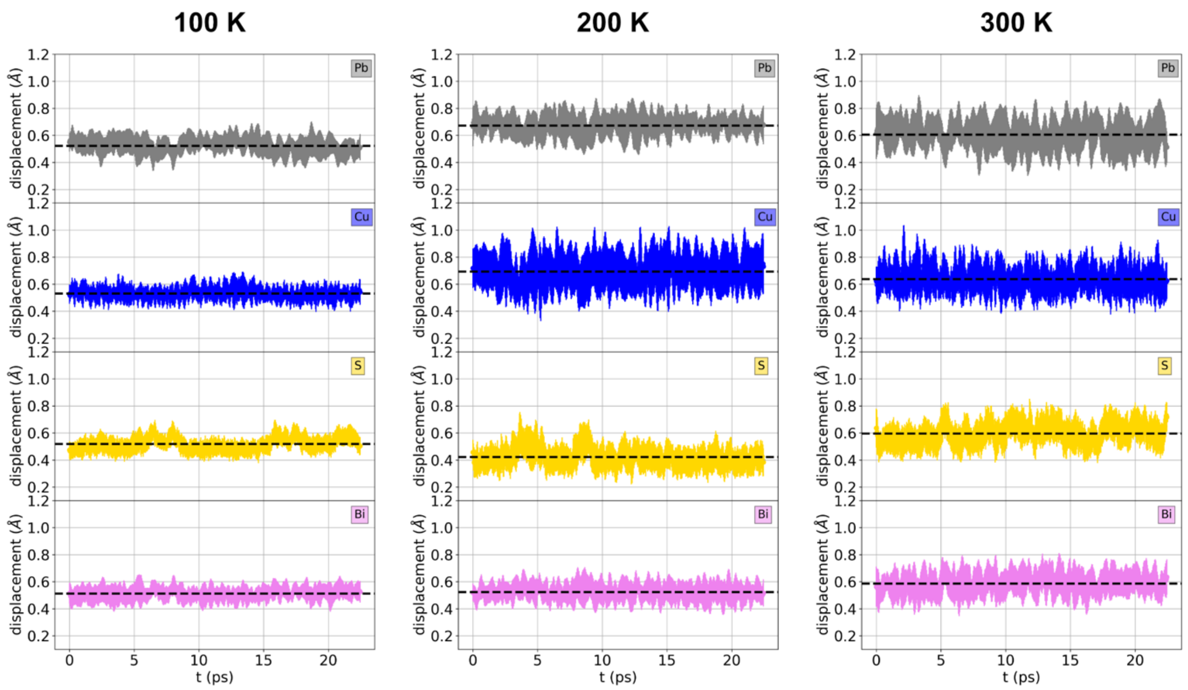

Figure S4. Ion displacements with respect to their average positions (black dotted line) computed over 23 ps, from the *ab initio* molecular dynamics trajectories at 100, 200, and 300 K. For each ion, the zero-displacement refers to the equilibrium position of the ion at 0 K.

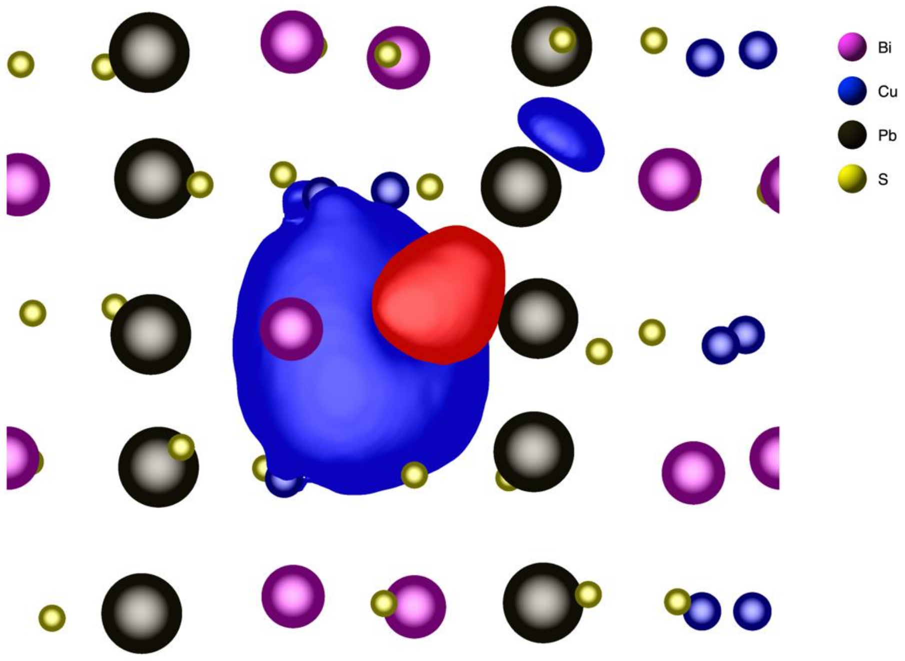

Figure S5. MLWF isosurface associated with the Pb^{2+} lone pair. Red and blue correspond to isovalues of -0.035 and 0.014, respectively.

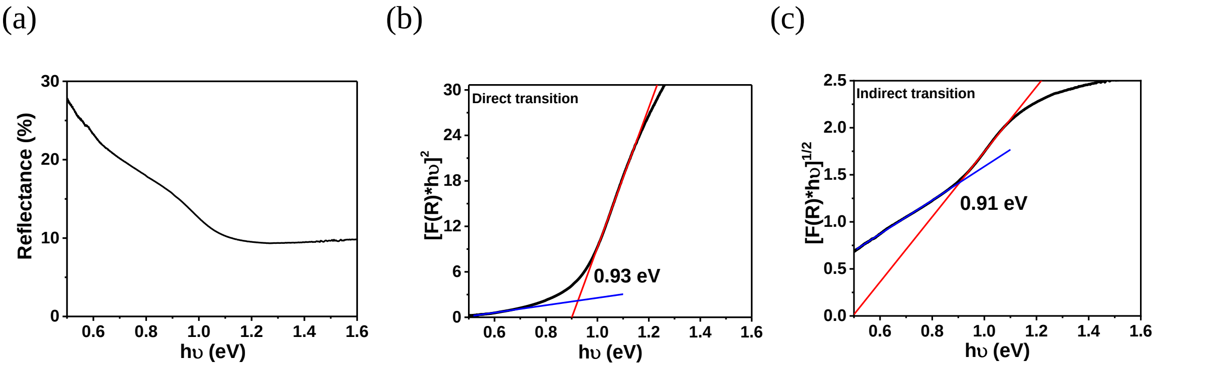

Figure S6. (a) Reflectance (%) as a function of photon energy, (b) evaluation of the direct band gap using $(F(R_\infty)h\nu)^2 = A(h\nu - E_g)$ and (c) evaluation of the indirect band gap using $(F(R_\infty)h\nu)^{1/2} = A(h\nu - E_g)$.

Table S6. Computed effective masses for aikinite.

k	m_1	m_2	m_3
Γ	5.91	17.46	1.06
X	7.42	9.14	5.97
	6.19	9.61	10.89
Z	20.10	-6.27	0.07
	-21.42	-7.12	-0.07
U	0.91	10.62	0.01
	0.84	11.19	-0.01

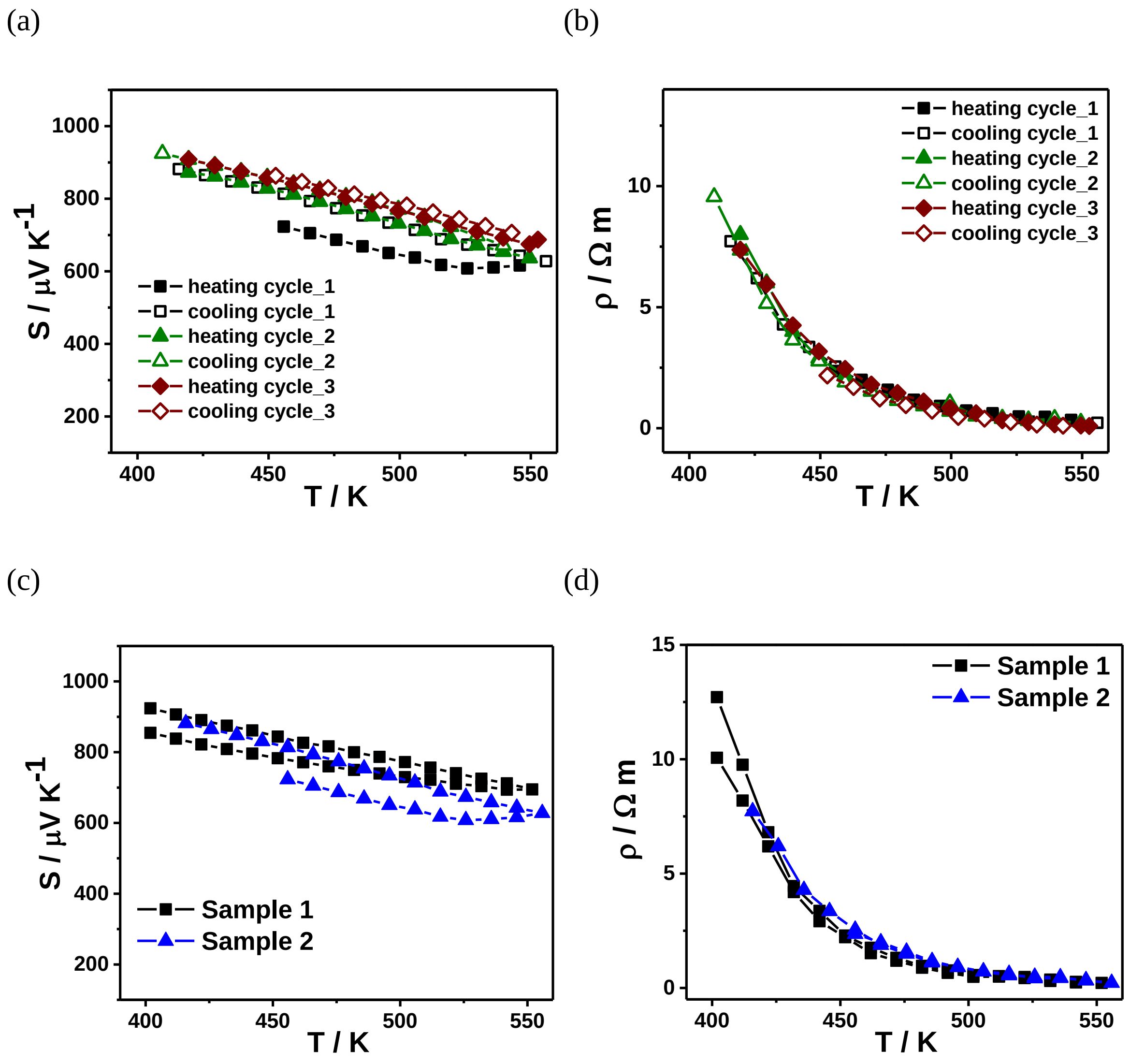

Figure S7. (a) Seebeck coefficient and (b) electrical resistivity as a function of temperature measured for three consecutive heating and cooling cycles for Sample 2; (c) Seebeck coefficient of two different batches with the same nominal composition and (d) electrical resistivity of two different batches of samples.

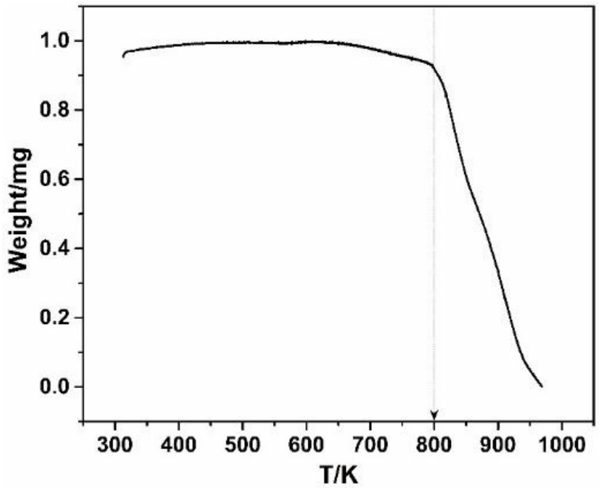

Figure S8. Thermogravimetric analysis (TGA) data on aikinite, $CuPbBiS_3$ (Sample 1). TGA measurements indicate that aikinite is thermally stable up to a temperature of 800 K

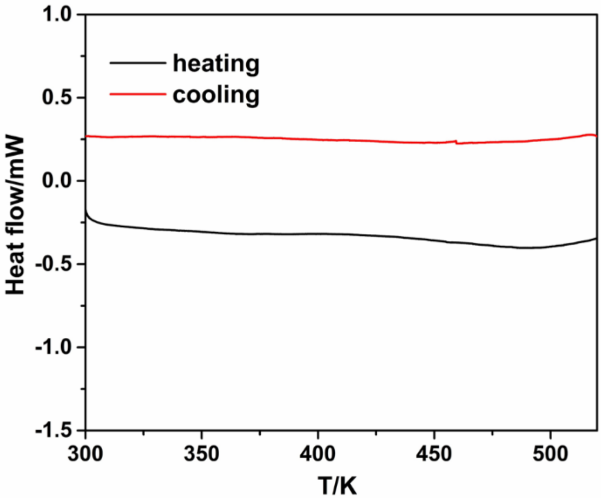

Figure S9. Differential scanning calorimetry (DSC) data for aikinite, $CuPbBiS_3$ (Sample 1). DSC does not show the occurrence of exothermic or endothermic peaks during the heating and/or cooling cycles.

(a)

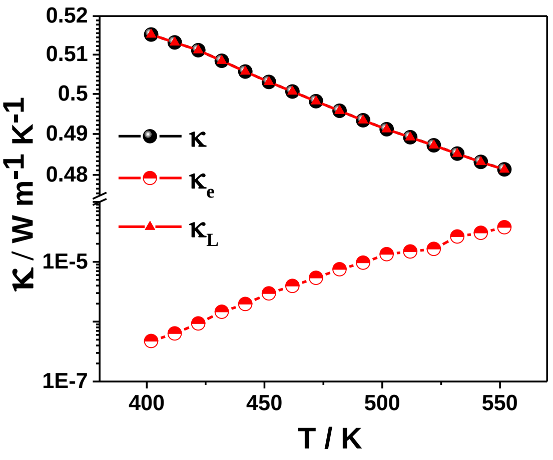

(b)

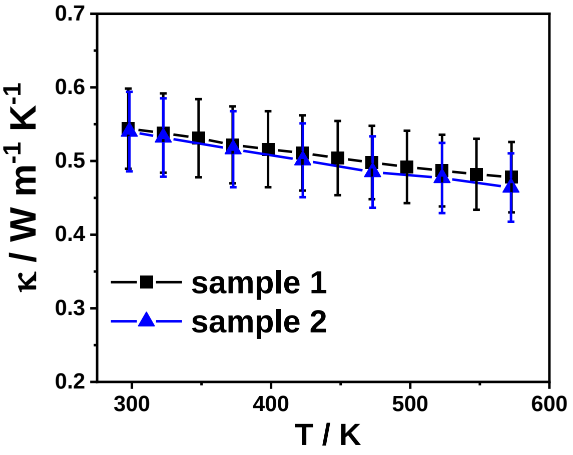

(c)

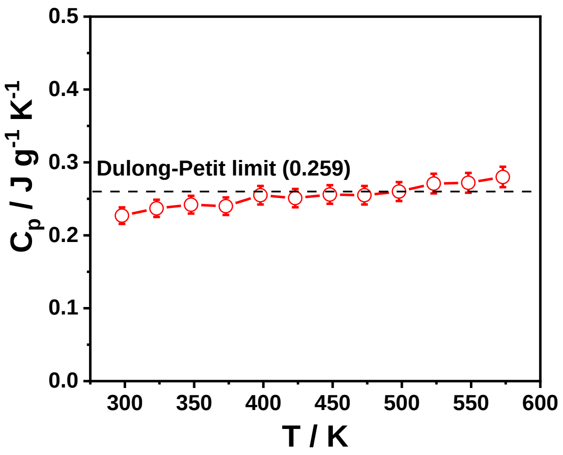

Figure S10. (a) Temperature dependence of the lattice thermal conductivity κ_L and electronic thermal conductivity κ_e along with the total thermal conductivity κ for Sample 1. (b) Thermal conductivity for two batches of $CuPbBiS_3$ with the same nominal composition. Error bars (10% uncertainty) are shown. (c) Heat capacity for Sample 1 measured on an LFA-447, using a pyroceram standard.

Table S7. Computed elastic constants for aikinite in GPa.

C_{11}	C_{22}	C_{33}	C_{44}	C_{55}	C_{66}	C_{12}	C_{13}	C_{23}
56.5	97.8	77.9	28.0	26.5	14.9	27.6	33.4	36.4

Table S8. Comparison of the sound velocity, Grüneisen parameter, Debye temperature and Young's modulus for a range of thermoelectric materials.

Material	Average sound velocity (m s^{-1})	Method of measurement	Grüneisen parameter	Debye temperature (K)	Young's modulus (GPa)	Ref.
$CuPbBiS_3$	1736	Ultrasonic Pulse echo method	1.59	183	42.7	This work
SnSe	1420	Ultrasonic Pulse echo method	3.13	142	-	9
In_4Se_3	2010	Ultrasonic Pulse echo method	1.5	198	47	10
Bi_2Te_3	1778	Continuous-wave resonance technique	1.5	164	-	11,12
PbTe	1924	Ultrasonic Pulse echo method	1.56	176.9	62.3	13
Mg_2Si	5350	Resonant ultrasound spectroscopy	1.19	570	110	14

Table S9. Comparison of previously reported values of the sound velocity, Grüneisen parameter and Young's modulus for materials in the $Cu_{1-x}\square_xPb_{1-x}Bi_{1+x}S_3$ series.

[19]Material	v_l / ms^{-1}	v_T / ms^{-1}	v_a / ms^{-1}	Grüneisen	Young's modulus (GPa)	Ref.
Bi_2S_3*	2409	1402	1555	1.47	27.6	15
Bi_2S_3	2864	1600	1781	1.62	41.5	16
Bi_2S_3*			1530	1.26	26.5	17
$CuPbBi_5S_9$			1758	1.92		18
$CuPbBi_5S_9$ (M)$	2732	1521	1694	1.6	37.8	19
$CuPbBi_5S_9$ (T)$	2511	1394	1553	1.64	27.1	19
$CuPbBi_5S_9$ (M+T)$	2761	1558	1733	1.58	41.4	19

*Measured on a porous sample; $ M, T, and M+T refer to the synthesis method. See Ref. 19.

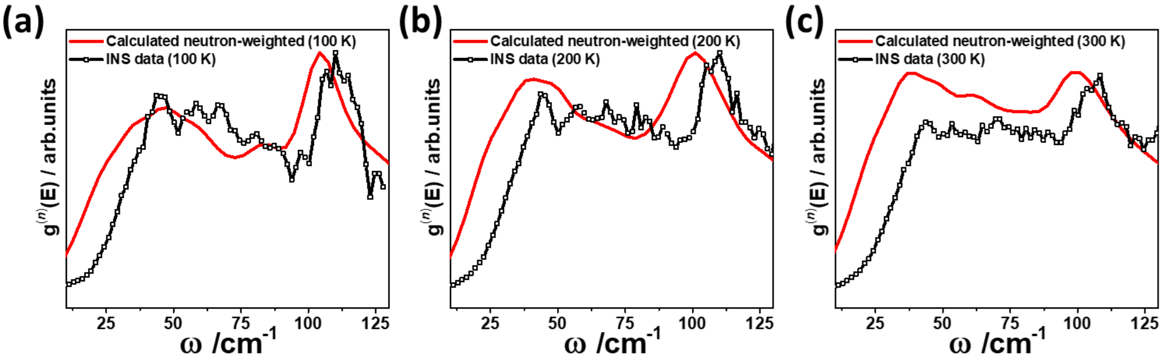

Figure S11. Comparison between the calculated neutron-weighted total DOS (convoluted with the instrumental resolution) and INS data at (a) 100 K, (b) 200 K and (c) 300 K.

Table S10. Temperature dependent shifts of the frequency of the six peaks found in the INS data.

T (K)	Centre of the phonon peak as obtained from Gaussian fits (cm^{-1})					
	Peak 1	Peak 2	Peak 3	Peak 4	Peak 5	Peak 6
10	31.7(4)	44.1(2)	59.1(2)	69.5(4)	80.648)	110.7(1)
100	36.5(4)	45.0(1)	56.2(3)	66.1(12)	84.0(5)	109.4(2)
200	36.9(3)	44.7(1)	56.6(3)	67.9(4)	81.5(4)	108.6(2)
300	37.3(2)	43.5(2)	55.8(3)	67.7(4)	83.1(3)	106.5(2)

Table S11. Temperature dependent changes in FWHM of the six peaks identified in the INS data.

T (K)	FWHM of the phonon peak as obtained from Gaussian fits (cm^{-1})					
	Peak 1 (Pb)	Peak 2	Peak 3	Peak 4	Peak 5	Peak 6 (Cu)
10	11.6(5)	13.3(8)	11.41(10)	8.8(8)	20.04(14)	12.7(3)
100	24.7(8)	7.2(5)	8.77(11)	18.34(25)	8.63(12)	11.5(4)
200	26.4(5)	6.1(5)	8.5(7)	14.01(14)	7.8(9)	10.2(5)
300	28.3(4)	5.4(6)	7.43(11)	17.38(11)	5.1(9)	8.7(6)

References:

(1) Baur, W. H. The Geometry of Polyhedral Distortions. Predictive Relationships for the Phosphate Group. *Acta Crystallogr. Sect. B* **1974**, *30* (5), 1195–1215. https://doi.org/10.1107/S0567740874004560.

(2) Robinson, K.; Gibbs, G. V.; Ribbe, P. H. Quadratic Elongation: A Quantitative Measure of Distortion in Coordination Polyhedra. *Science* **1971**, *172* (3983), 567–570. https://doi.org/10.1126/science.172.3983.567.

(3) Hoppe, R. Effective coordination numbers (ECoN) and mean fictive ionic radii (MEFIR). *Z. Für Krist. - Cryst. Mater.* **1979**, *150* (1–4), 23–52. https://doi.org/10.1524/zkri.1979.150.14.23.

(4) Siloi, I.; Gopal, P.; Curtarolo, S.; Nardelli, M. B.; Vaqueiro, P.; Fornari, M. Thermoelectric Properties of Minerals with the Mawsonite Structure. *ACS Appl. Energy Mater.* **2019**, *2* (11), 8068–8078. https://doi.org/10.1021/acsaem.9b01564.

(5) Lee, C.; Gonze, X. Ab Initio Calculation of the Thermodynamic Properties and Atomic Temperature Factors of SiO_2 α-Quartz and Stishovite. *Phys. Rev. B* **1995**, *51* (13), 8610–8613. https://doi.org/10.1103/PhysRevB.51.8610.

(6) Ali, M. A.; Nasir, M. T.; Khatun, M. R.; Islam, A. K. M. A.; Naqib, S. H. An Ab Initio Investigation of Vibrational, Thermodynamic, and Optical Properties of Sc_2AlC MAX Compound. *Chin. Phys. B* **2016**, *25* (10), 103102. https://doi.org/10.1088/1674-1056/25/10/103102.

(7) Allen, P. B.; Feldman, J. L.; Fabian, J.; Wooten, F. Diffusons, Locons, Propagons: Character of Atomic Vibrations in Amorphous Si. *Philos. Mag. B* **1999**, *79* (11–12), 1715–1731. https://doi.org/10.1080/014186399255836.

(8) Allen, P. B.; Feldman, J. L. Thermal Conductivity of Disordered Harmonic Solids. *Phys. Rev. B* **1993**, *48* (17), 12581–12588. https://doi.org/10.1103/PhysRevB.48.12581.

(9) Xiao, Y.; Chang, C.; Pei, Y.; Wu, D.; Peng, K.; Zhou, X.; Gong, S.; He, J.; Zhang, Y.; Zeng, Z.; Zhao, L.-D. Origin of Low Thermal Conductivity in SnSe. *Phys. Rev. B* **2016**, *94* (12), 125203. https://doi.org/10.1103/PhysRevB.94.125203.

(10) Luu, S. D. N.; Supka, A. R.; Nguyen, V. H.; Vo, D.-V. N.; T. Hung, N.; Wojciechowski, K. T.; Fornari, M.; Vaqueiro, P. Origin of Low Thermal Conductivity in In_4Se_3. *ACS Appl. Energy Mater.* **2020**, *3* (12), 12549–12556. https://doi.org/10.1021/acsaem.0c02489.

(11) Zhu, B.; Liu, X.; Wang, Q.; Qiu, Y.; Shu, Z.; Guo, Z.; Tong, Y.; Cui, J.; Gu, M.; He, J. Realizing Record High Performance in N-Type Bi_2Te_3-Based Thermoelectric Materials. *Energy Environ. Sci.* **2020**, *13* (7), 2106–2114. https://doi.org/10.1039/D0EE01349H.

(12) Jenkins, J. O.; Rayne, J. A.; Ure, R. W. Elastic Moduli and Phonon Properties of Bi_2Te_3. *Phys. Rev. B* **1972**, *5* (8), 3171–3184. https://doi.org/10.1103/PhysRevB.5.3171.

(13) Parashchuk, T.; Wiendlocha, B.; Cherniushok, O.; Knura, R.; Wojciechowski, K. T. High Thermoelectric Performance of P-Type PbTe Enabled by the Synergy of Resonance Scattering and Lattice Softening. *ACS Appl. Mater. Interfaces* **2021**, *13* (41), 49027–49042. https://doi.org/10.1021/acsami.1c14236.

(14) Castillo-Hernandez, G.; Yasseri, M.; Klobes, B.; Ayachi, S.; Müller, E.; de Boor, J. Room and High Temperature Mechanical Properties of Mg_2Si, Mg_2Sn and Their Solid Solutions. *J. Alloys Compd.* **2020**, *845*, 156205. https://doi.org/10.1016/j.jallcom.2020.156205.

(15) Guo, J.; Yang, J.; Ge, Z.-H.; Jiang, B.; Qiu, Y.; Zhu, Y.-K.; Wang, X.; Rong, J.; Yu, X.; Feng, J.; He, J. Realizing High Thermoelectric Performance in Earth-Abundant Bi_2S_3 Bulk Materials via Halogen Acid Modulation. *Adv. Funct. Mater.* **2021**, *31* (37), 2102838. https://doi.org/10.1002/adfm.202102838.

(16) Guo, J.; Ge, Z.-H.; Qian, F.; Lu, D.-H.; Feng, J. Achieving High Thermoelectric Properties of Bi_2S_3 via $InCl_3$ Doping. *J. Mater. Sci.* **2020**, *55* (1), 263–273. https://doi.org/10.1007/s10853-019-04008-3.

(17) Guo, J.; Wang, Z.-Y.; Chen, L.; Zhu, Y.-K.; Zhou, Y.; Shan, Q.; Feng, J.; Ge, Z.-H. $Bi_{0.33}(Bi_6S_9)Br$ Compositing in Bi_2S_3 Bulk Materials Forwards High Thermoelectric Properties. *Phys. Chem. Chem. Phys.* **2022**, *24* (39), 24290–24295. https://doi.org/10.1039/D2CP02805K.

(18) Maji, K.; Lemoine, P.; Renaud, A.; Zhang, B.; Zhou, X.; Carnevali, V.; Candolfi, C.; Raveau, B.; Al Rahal Al Orabi, R.; Fornari, M.; Vaqueiro, P.; Pasturel, M.; Prestipino, C.; Guilmeau, E. A Tunable Structural Family with Ultralow Thermal Conductivity: Copper-Deficient $Cu_{1-x}\square_xPb_{1-x}Bi_{1+x}S_3$. *J. Am. Chem. Soc.* **2022**, *144* (4), 1846–1860. https://doi.org/10.1021/jacs.1c11998.

(19) Liang, H.; Guo, J.; Zhou, Y.-X.; Wang, Z.-Y.; Feng, J.; Ge, Z.-H. $CuPbBi_5S_9$ Thermoelectric Material with an Intrinsic Low Thermal Conductivity: Synthesis and Properties. *J. Materiomics* **2022**, *8* (1), 174–183. https://doi.org/10.1016/j.jmat.2021.03.016.
